# Manifestations of Structural Racism and Inequities in Cardiovascular Health Across US Neighborhoods

**DOI:** 10.1001/jamahealthforum.2025.3864

**Published:** 2025-10-31

**Authors:** Wayne R. Lawrence, Hyokyoung G. Hong, Faustine Williams, Zachary Dyer, Nyahne Q. Bergeron, LaPrincess C. Brewer, Yingxi Chen, Denine R. Crittendon, Neal D. Freedman, Cameron B. Haas, Sarah S. Jackson, Connor D. Martz, Jennifer K. McGee-Avila, Cameron K. Ormiston, Catherine M. Pichardo, Charles R. Rogers, Eduardo J. Santiago-Rodríguez, Salma Shariff-Marco, Indira C. Turney, Tiffany M. Powell-Wiley, Wangjian Zhang, Meredith S. Shiels

**Affiliations:** 1 Division of Cancer Epidemiology and Genetics, National Cancer Institute, National Institutes of Health, Rockville, Maryland; 2 Division of Intramural Research, National Institute on Minority Health and Health Disparities, National Institutes of Health, Bethesda, Maryland; 3 Department of Population and Quantitative Health Sciences, UMass Chan Medical School, Worcester, Massachusetts; 4 Department of Cardiovascular Medicine, Mayo Clinic, Rochester, Minnesota; 5 Center for Clinical and Translational Science, Mayo Clinic, Rochester, Minnesota; 6 Division of Cancer Control and Population Sciences, National Cancer Institute, National Institutes of Health, Rockville, Maryland; 7 Department of Population Health Sciences, College of Medicine, University of Central Florida, Orlando; 8 Department of Medical Education, Icahn School of Medicine at Mount Sinai, New York, New York; 9 Division of Community Health and Population Science, National Institute on Minority Health and Health Disparities, National Institutes of Health, Bethesda, Maryland; 10 Men’s Health Inequities Research Lab, Milwaukee, Wisconsin; 11 Department of Epidemiology and Biostatistics, School of Medicine, University of California San Francisco, San Francisco; 12 Helen Diller Family Comprehensive Cancer Center, University of California San Francisco, San Francisco; 13 Laboratory of Epidemiology and Population Sciences, National Institute on Aging, National Institutes of Health, Baltimore, Maryland; 14 Cardiovascular Branch, Division of Intramural Research, National Heart, Lung, and Blood Institute, National Institutes of Health, Bethesda, Maryland; 15 Department of Medical Statistics, School of Public Health, Sun Yat-sen University, Guangzhou, China; 16 Department of Environmental Health Sciences, College of Integrated Health Science, State University of New York at Albany, Albany

## Abstract

**Importance:**

The prevalence of cardiovascular disease differs substantially across neighborhoods. However, few studies have examined the contribution of neighborhood-level structural racism to inequities in cardiovascular health.

**Objective:**

To investigate the association between neighborhood-level manifestations of structural racism and the prevalence of cardiovascular clinical and behavioral risk factors and cardiovascular diseases.

**Design, Setting, and Participants:**

This cross-sectional study consisted of 71 915 US census tracts. The Structural Racism Effect Index (SREI) was appended to the 2020 Centers for Disease Control and Prevention’s PLACES dataset. Race and ethnicity were self-reported and obtained from the 2019 American Community Survey 5-year estimates. Data analysis was conducted from June 2024 to October 2024.

**Exposure:**

The SREI is a composite score of census tract measures within 9 domains affected by structural racism (built environment, criminal justice, education, employment, housing, income and poverty, social cohesion, transportation, and wealth). The SREI scores were categorized into distribution-based quintiles, where higher quintiles represented greater neighborhood structural racism.

**Main Outcomes and Measures:**

Neighborhood-level prevalence of cardiovascular clinical (high blood pressure, high cholesterol, obesity, and diabetes) and behavioral (current cigarette smoking and no leisure-time physical activity) risk factors and cardiovascular disease (coronary heart disease and stroke). Multilevel-linear mixed models, adjusting for confounders, were used to estimate adjusted prevalence rate ratios (aPRR).

**Results:**

Among 71 915 census tracts, neighborhoods in the highest quintiles of structural racism were more likely than neighborhoods in the lowest quintile to be in the southern region (quintile 1, 19.9% vs quintile 5, 56.8%). The proportion of the population that was Black was largest in neighborhoods in the highest quintile of structural racism (quintile 1, 2.2% vs quintile 5, 23.0%). After adjustment, compared with the lowest quintile of structural racism, the highest quintile had significantly higher prevalence rates of cardiovascular clinical risk factors, including high blood pressure (aPRR, 1.37; 95% CI, 1.37-1.38), obesity (aPRR, 1.40; 95% CI, 1.39-1.40), and diabetes (aPRR, 1.80; 95% CI, 1.79-1.81). Similarly, for cardiovascular behavioral risk factors, the highest quintile of structural racism had significantly higher prevalence rates of current cigarette smoking (aPRR, 1.84; 95% CI, 1.83-1.85) and no leisure-time physical activity (aPRR, 1.88; 95% CI, 1.87-1.89). Additionally, for cardiovascular diseases, the highest quintile of structural racism had higher prevalence rates of coronary heart disease (aPRR, 1.80; 95% CI, 1.79-1.82) and stroke (aPRR, 1.99; 95% CI, 1.98-2.00).

**Conclusions and Relevance:**

In this cross-sectional study, structural racism was associated with inequities in neighborhood cardiovascular health, highlighting opportunities for place-based prevention efforts.

## Key Points

**Question:**

Across US neighborhoods, are neighborhood-level manifestations of structural racism associated with inequities in cardiovascular health?

**Findings:**

In this cross-sectional study of 71 915 census tracts, neighborhoods in the highest quintile of structural racism experienced significantly higher prevalence rates of cardiovascular clinical (high blood pressure, obesity, and diabetes) and behavioral (cigarette smoking and no leisure-time physical activity) risk factors and cardiovascular diseases (coronary heart disease and stroke) compared with those in the lowest quintile. Similar results were observed for each domain of structural racism (eg, housing, education, and employment).

**Meaning:**

Findings show that structural racism should be considered when developing health equity–oriented, placed-based interventions.

## Teaser

This cross-sectional study examines the association of neighborhood-level structural racism with the prevalence of cardiovascular clinical and behavioral risk factors and cardiovascular diseases in the US.

## Introduction

Cardiovascular disease is the leading cause of morbidity and mortality among adults in the US,^1,2,3^ with racial inequities persisting despite advances in prevention, management, and treatment. Although racism has been identified as a contributor to racial inequities in cardiovascular health, the explicit examination of the multidimensional manifestations of structural racism remains limited.^4,5,6^

Structural racism refers to the ways in which society perpetuates racial discrimination through intersecting and mutually reinforcing inequitable systems, which bolster discriminatory laws, policies, practices, and norms, leading to a differential distribution of resources and opportunities.^7,8^ Structural racism operates through residential segregation, creating racialized neighborhoods with differential socioeconomic conditions, which are further shaped by historical and ongoing discriminatory practices.^4,9^ This structural racism results in neighborhoods with predominantly racially and/or ethnically minoritized residents experiencing targeted economic and political exclusion, and greater adverse health conditions.^4,10,11^ Most research on the impact of structural racism on cardiovascular health has focused on a singular component (eg, residential segregation). Given the systemic nature of racism, scholars have recommended the use of multidimensional measures that capture manifestations of structural racism across numerous levels and institutions.^8,12,13^

In this study, we aim to address these gaps by evaluating the associations between neighborhood-level manifestations of structural racism and the prevalence of cardiovascular clinical and behavioral risk factors and cardiovascular diseases. We hypothesized that greater neighborhood-level manifestations of structural racism would be associated with increased prevalence rates of cardiovascular risk factors and cardiovascular diseases.

## Methods

This cross-sectional study followed the Strengthening the Reporting of Observational Studies in Epidemiology (STROBE) reporting guidelines.^14^ The National Institutes of Health institutional review board waived approval and informed consent because the study used publicly available deidentified data.

### Structural Racism

Structural racism was measured using the Structural Racism Effect Index (SREI) created by Dyer and colleagues.^15^ Briefly, the SREI is a validated multidimensional census tract–level composite score of geographic sociopolitical-economic indicators that reflect manifestations of structural racism across 9 interconnected domains (built environment, criminal justice, education, employment, housing, income and poverty, social cohesion, transportation, and wealth). The list of geographic variables included in each domain is shown in eTable 1 in Supplement 1. All variables are oriented in the same direction, so that higher values indicate greater structural disadvantages. Each variable is standardized, then the standardized values are summed in each domain, and then the sum is restandardized. The final SREI measure is operationalized as the sum of each domain score, with population-weighted values standardized to reflect a mean of 0 and SD of 1. For the SREI and its domains, positive scores indicate greater negative effects of structural racism, and negative scores indicate fewer negative effects. The SREI is calculated for all US census tracts with at least 100 households, excluding those with missing data either for all variables in any domain or more than 9 variables total. SREI scores were categorized into distribution-based quintiles, where higher quintiles represented greater negative effects of structural racism.

### Cardiovascular Risk Factors and Cardiovascular Diseases

Census tract prevalence of cardiovascular clinical risk factors (high blood pressure, high cholesterol, diabetes, and obesity), cardiovascular behavioral risk factors (current cigarette smoking and no leisure-time physical activity), and cardiovascular diseases (coronary heart disease and stroke) among adults aged 18 years or older were extracted from the 2020 Centers for Disease Control and Prevention (CDC) PLACES dataset, which is based on the 2017-2018 Behavior Risk Factor Surveillance System (BRFSS).^16^ Details on each outcome are shown in eTable 2 in Supplement 1. CDC PLACES provides census tract estimates of health risk behaviors and outcomes from BRFSS, based on multilevel regression and poststratification approaches for small-area estimation. Validation studies have demonstrated at different area levels strong concordance between model-based estimates and direct survey estimates.^17,18^

### Covariates

Census tract median age, proportion of female adults, proportion of uninsured adults, and racial and ethnic composition were obtained from the 2019 American Community Survey 5-year estimates. A principle of this study is that race represents a sociopolitical construct (ie, not genetic or biological) primarily based on skin complexion, ethnicity, and social differentiation that disadvantages some groups while advantaging others.^19,20,21,22^ The census tract percentage of adults reporting visits to a physician for routine checkup within the past year was obtained from the 2020 CDC PLACES. The number of nonfederal primary care physicians and physicians specializing in cardiovascular disease per 100 000 population at the county level was extracted from the 2018 Area Health Resources File. Metropolitan status was based on the 2013 Rural-Urban Continuum Codes from the US Department of Agriculture, and regions were based on the US Census Bureau.

### Statistical Analyses

Data analysis was conducted from June 2024 to October 2024. Descriptive analyses summarized population characteristics across quintiles of structural racism by their frequency and distribution. Guided by prior research and conceptual framework on racism and health, in the main analysis, we calculated the adjusted prevalence rate ratio (aPRR) of each quintile group compared with the first quintile group, with multilevel linear mixed models weighted for census tract population size, clustered at the county level, and adjusted for age, proportion of female adults, percentage of the population that was non-Hispanic White, metropolitan status, region, number of cardiovascular disease physicians per 100 000 population, number of primary care physicians per 100 000 population, percentage of adults having received a routine checkup in the last year, and percentage of uninsured adults.^22,23,24^ The analysis was repeated to evaluate the associations between each domain of structural racism (in distribution-based quintiles) and cardiovascular risk factors and diseases.

We further evaluated whether associations between census tract–level structural racism and cardiovascular risk factors and diseases varied by distribution-based quartiles (quartile 1 = low and quartile 4 = high) for each racial and ethnic group (non-Hispanic Asian American, non-Hispanic Black, non-Hispanic White, and Hispanic or Latino). Stratified analyses were performed by quartile of each racial and ethnic group to evaluate associations between quintiles of structural racism and aPRR of cardiovascular risk factors and diseases. Interactions were examined using likelihood ratio tests by comparing the model fit with and without interaction terms. Analysis was performed using R, version 4.3.0 (R Project for Statistical Computing) and SAS, version 9.4 (SAS Institute). Two-sided *P* < .05 was considered statistically significant.

### Sensitivity Analyses

We performed 2 sensitivity analyses. In the first sensitivity analysis, based on prior studies, cigarette smoking can be considered a confounder or mediator.^25,26,27,28^ Therefore, we repeated the main analysis for cardiovascular clinical risk factors and diseases, adjusting for current cigarette smoking. In the second sensitivity analysis, we did not adjust for census tract–level percentage of the population that was non-Hispanic White in the main analysis.

To address the possibility that unmeasured factors, including ecological confounding, contributed to observed associations in the main analysis, we performed exact geospatial matching of census tracts that were geospatially close but at the extremes of structural racism (quintile 5 vs quintile 1). Specifically, we performed caliper matching on geospatial proximity (within 50 miles), proportion of female adults (within 5%), age composition (within 5% of the total percentage of adults aged ≥65 years), per capita rate of cardiovascular disease physicians (within 5 per 100 000 population), per capita rate of primary care physicians (within 10 per 100 000 population), and percentage of adults having received a routine checkup in the last year (within 5%).^23,29^ Geographic distance was calculated using longitude and latitude coordinates of the geometric centroid of census tracts. Multilevel linear mixed modeling was conducted, incorporating census tract pair random effects, to assess associations between structural racism and cardiovascular risk factors and diseases.

## Results

### Neighborhood Characteristics

**Figure 1.**
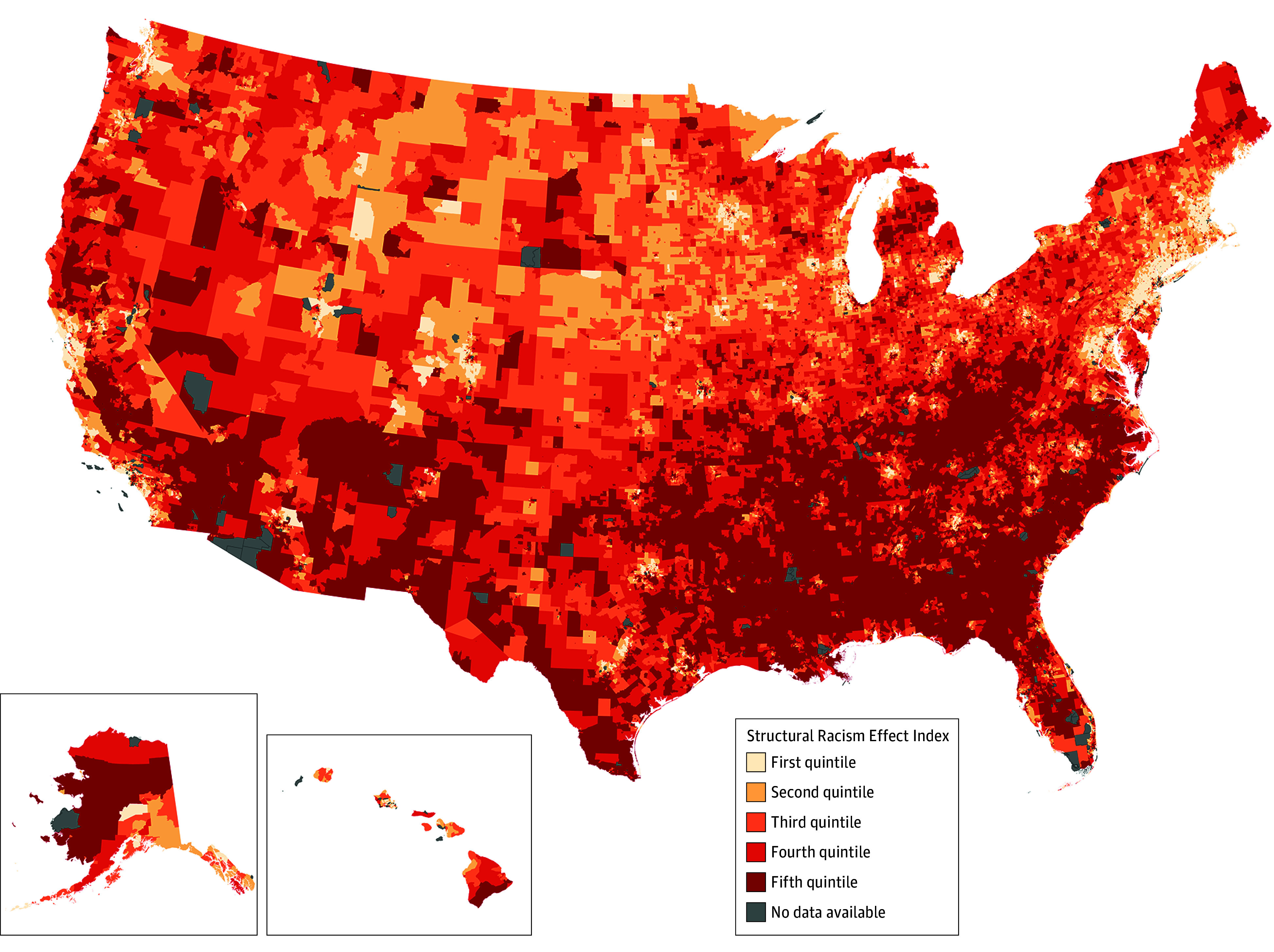
Neighborhood Structural Racism in the US The Structural Racism Effect Index includes a score for 71 915 US census tracts. Higher quintiles indicate greater negative effects of structural racism.

This study included a total of 71 915 census tracts (hereafter, neighborhoods), representing 97% of the total US census tracts. The geographic distribution of neighborhood-level structural racism is presented in Figure 1. Compared with the lowest quintile of structural racism, neighborhoods in the highest quintile were more likely to be in the southern region (56.8%) and metropolitan areas (75.0%) and had the highest median percentage of uninsured individuals (12.5%) (Table 1). The proportion of the population that was Black was highest in neighborhoods in the highest quintile of structural racism (quintile 1, 2.2% vs quintile 5, 23.0%). Compared with the lowest quintile of structural racism, the highest quintile had a higher mean prevalence for all cardiovascular risk factors and diseases (eTable 3 in Supplement 1).

**Table 1.** Characteristics of Neighborhoods by Structural Racism Quintile^a^

Characteristic	All neighborhoods (N = 71 915)	Q1 [lowest] (n = 14 383)	Q2 (n = 14 383)	Q3 (n = 14 384)	Q4 (n = 14 383)	Q5 [highest] (n = 14 382)
Total population, No.	307 715 963	64 214 812	63 719 278	62 726 652	62 021 058	55 034 163
Age, median (IQR), y	39.3 (34.4-44.4)	42.4 (38.0-46.4)	40.6 (36.4-45.4)	39.7 (35.2-44.7)	37.8 (33.3-43.1)	35.0 (30.5-40.4)
Sex, median (IQR), %						
Female	50.9 (48.8-53.1)	50.9 (49.1-52.7)	50.8 (48.9-52.8)	50.7 (48.7-52.8)	50.8 (48.6-53.1)	51.3 (48.6-54.2)
Male	49.1 (46.9-51.2	49.1 (47.3-50.9)	49.2 (47.2-51.1)	49.3 (47.2-51.3)	49.2 (46.9-51.4)	48.7 (45.8-51.4)
Racial composition, median (IQR), %^b^						
Asian American	1.6 (0.3-5.4)	5.8 (2.3-13.1)	2.4 (0.7-6.6)	1.4 (0.3-4.4)	0.9 (0.0-2.9)	0.3 (0.0-1.5)
Black	4.3 (1.0-15.6)	2.2 (0.7-5.2)	2.9 (0.7-8.5)	3.5 (0.7-11.8)	6.0 (1.2-18.9)	23.0 (5.2-58.0)
White	80.6 (59.8-91.9)	84.4 (72.2-91.8)	86.1 (71.1-93.9)	84.9 (67.6-94.0	78.9 (58.6-91.6)	56.8 (27.6-78.6)
Hispanic or Latino ethnicity, median (IQR), %	7.6 (2.8-20.9)	6.5 (3.2-12.1)	7.1 (2.8-17.6)	7.8 (2.7-21.7)	9.8 (2.8-32.6)	9.1 (2.2-39.6)
Racially and ethnically minoritized population composition, median (IQR), %^c^	30.8 (13.2-62.2)	22.7 (12.8-37.7)	21.7 (9.7-43.8)	24.3 (9.6-50.5)	36.7 (14.0-68.4)	70.4 (38.5-91.5)
Uninsured population, median (IQR), %^d^	6.7 (3.6-11.7)	2.8 (1.5-4.7)	5.0 (3.1-7.9)	6.9 (4.4-10.7)	9.7 (6.2-14.5)	12.5 (8.0-18.6)
Health care access, median (IQR)						
Rate of annual physician visit, %^e^	76.3 (72.5-79.1)	76.6 (72.8-79.8)	76.0 (72.4-78.8)	76.0 (72.4-78.4)	75.9 (72.1-78.2)	77.0 (73.1-80.5)
Primary care physicians per 100 000 population^f^	71.9 (55.5-94.2)	91.2 (71.9-112.8)	74.4 (59.9-94.2)	70.9 (53.4-88.2)	66.6 (47.6-82.6)	67.6 (46.6-84.9)
Cardiologists per 100 000 population^g^	6.3 (3.1-9.6)	7.9 (5.6-12.6)	6.1 (3.5-9.3)	5.3 (2.5-8.3)	5.0 (1.9-8.0)	5.9 (2.3-9.5)
Metropolitan status, No. (%)^h^						
Metropolitan	59 958 (83.4)	14 068 (97.8)	12 817 (89.1)	11 546 (80.3)	10 742 (74.7)	10 785 (75.0)
Nonmetropolitan	11 957 (16.6)	315 (2.2)	15,66 (10.9)	2838 (19.7)	3641 (25.3)	3597 (25.0)
US Census Bureau region, No. (%)^i^						
Midwest	16 897 (23.5)	2799 (19.5)	3929 (27.3)	4073 (28.3)	3169 (22.0)	2927 (20.3)
Northeast	13 272 (18.5)	4576 (31.8)	3308 (23.0)	2486 (17.3)	1548 (10.8)	1354 (9.4)
Pacific	469 (0.6)	152 (1.1)	180 (1.3)	83 (0.6)	45 (0.3)	9 (0.1)
South	25 886 (36.0)	2864 (19.9)	3555 (24.7)	4541 (31.6)	6756 (47.0)	8170 (56.8)
West	15 391 (21.4)	3992 (27.7)	3411 (23.7)	3201 (22.2)	2865 (19.9)	1922 (13.4)

Abbreviation: Q, quintile.

^a^ Higher quintiles indicate greater negative effects of structural racism.

^b^ Due to small counts, American Indian or Alaska Native and Native Hawaiian or Other Pacific Islander racial groups are not reported.

^c^ Percentage of census tract composed of racial and ethnic minority individuals (all persons except non-Hispanic White individuals).

^d^ Census tract-level percentage of civilian and noninstitutionalized population with no health insurance.

^e^ Census tract-level measure from the Centers for Disease Control and Prevention’s PLACES dataset of the percentage of adults aged ≥18 years who report having been to a doctor for a routine checkup (eg, a general physical examination, not an examination for a specific injury, illness, or condition) in the previous year.

^f^ County-level number of nonfederal primary care physicians per 100 000 population from the Area Health Resources File.

^g^ County-level number of physicians specializing in cardiovascular disease per 100 000 population from the Area Health Resources File.

^h^ Derived from 2013 Rural-Urban Continuum Codes from the US Department of Agriculture.

^i^ Derived from US Census Bureau.

### Structural Racism

**Figure 2.**
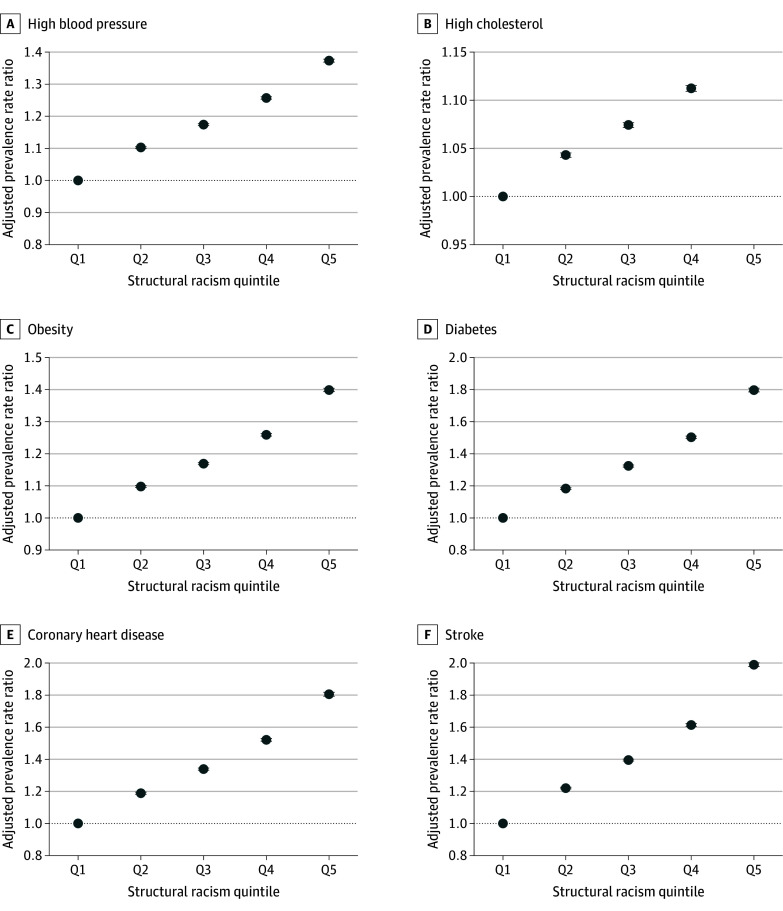
Adjusted Prevalence Rate Ratios for Cardiovascular Clinical Risk Factors and Cardiovascular Diseases by Structural Racism Quintile Multilevel linear mixed models weighted for population size and clustered at the county level, adjusting for age (median age), sex (percentage of female adults), percentage of non-Hispanic White population, metropolitan status, US Census Bureau region (Midwest, Northeast, Pacific, South, and West), number of cardiovascular disease physicians per 100 000 population, number of primary care physicians per 100 000 population, percentage of adults having received a routine checkup in the last year, and percentage of uninsured adults. Higher quintiles indicate greater negative effects of structural racism. Whiskers represent 95% CI. Q1 represents reference group. Q indicates quintile.

In the unadjusted model, greater structural racism was significantly associated with a higher prevalence rate ratio of cardiovascular risk factors and diseases (eTable 4 in Supplement 1). After adjustment, compared with the lowest quintile of structural racism, the prevalence rate for diabetes was higher in the highest quintile (aPRR, 1.80; 95% CI, 1.79-1.81), and the prevalence rate for obesity was also higher (aPRR, 1.40; 95% CI, 1.39-1.40) (Figure 2). Similar patterns were observed for high cholesterol (aPRR, 1.16; 95% CI, 1.16-1.17) and high blood pressure (aPRR, 1.37; 95% CI, 1.37-1.38). For cardiovascular diseases, after adjustment, the highest quintile of structural racism had higher prevalence rates for coronary heart disease (aPRR, 1.80; 95% CI, 1.79-1.82) and stroke (aPRR, 1.99; 95% CI, 1.98-2.00) compared with the lowest quintile. For behavioral risk factors, compared with the lowest quintile of structural racism, the highest quintile had a higher prevalence rate for cigarette smoking (aPRR, 1.84; 95% CI, 1.83-1.85) and no leisure-time physical activity (aPRR, 1.88; 95% CI, 1.87-1.89) (eFigure 1 in Supplement 1).

### Domains of Structural Racism

Compared with the lowest quintile for the employment domain, the highest quintile was associated with high blood pressure (aPRR, 1.21; 95% CI, 1.21-1.22). Similarly, for the built environment domain, compared to the lowest quintile, the highest quintile was associated with high cholesterol (aPRR, 1.10; 95% CI, 1.10-1.11) (eTable 5 in Supplement 1). For the wealth domain, compared with the lowest quintile, the highest quintile was associated with an increased prevalence rate for obesity (aPRR, 1.26; 95% CI, 1.26-1.27) and diabetes (aPRR, 1.53; 95% CI, 1.52-1.54). Similar results for the wealth domain were observed for coronary heart disease (aPRR, 1.60; 95% CI, 1.59-1.61) and stroke (aPRR, 1.71; 95% CI, 1.70-1.73). For the criminal justice domain, current cigarette smoking had the largest prevalence rate (aPRR, 1.05; 95% CI, 1.03-1.06) (eTable 6 in Supplement 1). However, across cardiovascular risk factors and diseases, the highest prevalence rates were observed for the education domain and the income and poverty domain, with stroke having the highest rate (education: aPRR, 1.87 [95% CI, 1.86-1.88]; income and poverty: aPRR, 1.85 [95% CI, 1.84-1.87]).

### Stratified Analysis by Neighborhood Racial and Ethnic Composition

In analyses stratifying neighborhoods based on the proportion of Asian American residents, in the fourth quartile, the highest quintile of structural racism had a higher prevalence rate for no leisure-time physical activity, compared with the lowest quintile (aPRR, 1.98; 95% CI, 1.95-2.00; *P* < .001 for interaction) (eTable 7 in Supplement 1). In neighborhoods with the highest proportion of Black residents, the highest quintile of structural racism had a 42% higher prevalence rate for obesity, compared with the lowest quintile (aPRR, 1.42; 95% CI, 1.41-1.43; *P* < .001 for interaction) (eTable 8 in Supplement 1). Similarly, in neighborhoods with the highest proportion of Hispanic or Latino residents, the highest quintile of structural racism had a higher prevalence rate for diabetes (aPRR, 1.91; 95% CI, 1.89-1.94; *P* < .001 for interaction) (eTable 9 in Supplement 1). Finally, in neighborhoods with the highest proportion of White residents, the highest quintile of structural racism had a higher prevalence rate for coronary heart disease compared with the lowest (aPRR, 1.71; 95% CI, 1.69-1.73; *P* < .001 for interaction) (eTable 10 in Supplement 1).

### Sensitivity Analyses

The associations between structural racism and cardiovascular clinical risk factors and diseases remained after including current cigarette smoking prevalence in the adjusted model (eFigure 2 in Supplement 1). In the second sensitivity analysis, removing the percentage of the population that was non-Hispanic White from the model, the results were similar to the main analysis for cardiovascular clinical risk factors and diseases (eFigure 3 in Supplement 1) and cardiovascular behavioral risk factors (eFigure 4 in Supplement 1). The geospatial- and covariate-based matching of neighborhoods at the highest and lowest extremes of structural racism identified 6474 match pairs (Table 2). After adjustment, compared with quintile 1, quintile 5 had a larger prevalence rate for all cardiovascular risk factors and diseases. For behavioral and clinical risk factors, the largest prevalence rate was observed for current cigarette smoking (aPRR, 1.97; 95% CI, 1.94-2.01) and diabetes (aPRR, 1.93; 95% CI, 1.90-1.96). For cardiovascular disease, stroke had the largest prevalence rate (aPRR, 2.06; 95% CI, 2.03-2.09).

**Table 2.** Adjusted Prevalence Rate Ratios for Cardiovascular Risk Factors and Cardiovascular Disease Among Structural Racism Matched Neighborhoods^a^

Variable	Geospatial and covariate-matched neighborhoods (n = 6474)^b^		
	Mean (SD) disease prevalence, %		Adjusted prevalence rate ratio (95% CI)
	Lowest structural racism (quintile 1)^c^	Highest structural racism (quintile 5)^d^	
Cardiovascular behavioral risk factors			
Current cigarette smoking	11.9 (2.5)	24.1 (4.6)	1.97 (1.94-2.01)
No leisure time physical activity	16.7 (3.1)	35.7 (5.4)	1.95 (1.93-1.98)
Cardiovascular clinical risk factors			
High blood pressure	26.1 (5.0)	37.2 (7.4)	1.42 (1.41-1.43)
High cholesterol	30.7 (4.4)	33.5 (3.7)	1.21 (1.20-1.22)
Obesity	25.7 (3.9)	39.5 (5.1)	1.49 (1.47-1.50)
Diabetes	7.6 (1.7)	15.8 (3.8)	1.93 (1.90-1.96)
Cardiovascular diseases			
Coronary heart disease	4.6 (1.3)	7.8 (2.1)	1.99 (1.96-2.03)
Stroke	2.2 (0.5)	4.8 (1.4)	2.06 (2.03-2.09)

^a^ Multilevel linear mixed models weighted for population size and clustered at the county level, adjusting for age (median age), sex (percentage of female adults), percentage of non-Hispanic White population, metropolitan status, US Census Bureau region (Midwest, Northeast, Pacific, South, and West), health care access (number of cardiovascular disease physicians per 100 000 population, number of primary care physicians per 100 000 population, percentage of adults having received a routine checkup in the last year, and percentage of uninsured adults).

^b^ Highest and lowest structural racism quintile were exact matched on metropolitan status and region, and caliper matched on geospatial proximity (within 50 miles), sex composition (within 5%), age composition (within 5% of the total percentage of adults aged ≥65 years), per capita rate of cardiovascular disease physicians (within 5 per 100 000 population), per capita rate of primary care physicians (within 10 per 100 000 population), and percentage of adults having received a routine checkup in the last year (within 5%).

^c^ Lowest quintile of structural racism. Lowest structural racism (quintile 1) was the reference group.

^d^ Highest quintile of structural racism.

## Discussion

In this cross-sectional study across US neighborhoods, greater neighborhood-level manifestations of structural racism were consistently associated with higher prevalence of neighborhood cardiovascular risk factors and diseases. This trend held across individual domains of structural racism for clinical risk factors, behavioral risk factors, and cardiovascular diseases.

Neighborhood patterns of cardiovascular risk factors and diseases are well documented.^9,30^ Our results extend this documentation by demonstrating that neighborhood-level factors that represent manifestations of structural racism are a significant determinant of cardiovascular health. Adverse neighborhood conditions can harm cardiovascular health through long-term physiological and behavioral changes as well as reduced access to health-promoting resources.^31,32,33^ Residents in neighborhoods with more socioenvironmental stressors often experience excessive activation of the stress response system, increasing risks for insulin resistance, metabolic syndrome, reduced glucose regulation, and elevated blood pressure and cortisol levels.^34,35,36,37,38^ Studies also show that such socioenvironmental adversity increases harmful coping behaviors, such as cigarette smoking and physical inactivity, both of which are associated with cardiovascular risk factors and diseases.^39,40,41^ In the present study, higher structural racism was associated with greater cigarette smoking and no leisure-time physical activity. Racialized tobacco marketing, including targeted price discounts (eg, multipack cigarette coupons) in neighborhoods with predominantly Black residents might further contribute to smoking.^28^ The physiological and behavioral impacts of neighborhood structural disadvantage likely explain the strong link between greater structural racism and cardiovascular risk factors and diseases. Similar patterns were observed across all domains of structural racism when evaluated independently, with the strongest associations in the education and the income and poverty domains. A potential explanation is that neighborhoods with limited high-quality education opportunities and/or low-income often lack health-promoting resources and experience greater barriers in accessing specialty medical care.^10,27,30^

Our findings showed that, regardless of the racial and ethnic composition, greater structural racism was associated with higher prevalence rates of cardiovascular risk factors and diseases. However, the strongest associations differed across quartiles of neighborhood racial and ethnic composition. For example, the prevalence rate ratio for the association between structural racism and obesity was greatest in neighborhoods with the highest proportion of Asian American, Black, and Hispanic or Latino residents, while in contrast, the prevalence rate ratio was greatest in neighborhoods with the lowest proportion of White residents. These results suggest that the association between neighborhood structural racism and cardiovascular risk factors and diseases is potentially moderated by racial and ethnic composition.

Several indices that measure area-level social determinants of health risk (eg, area deprivation index and social vulnerability index) have linked greater disadvantage to poor cardiovascular health.^23,42,43^ The SREI is distinguished by its comprehensive approach to capturing the breadth and depth of how structural racism manifests disadvantage across institutional domains.^44^ For example, while most indices rely on broad indicators such as educational attainment and unemployment rates, the SREI incorporates additional, domain-specific measures—such as per-pupil education spending and retail job availability—to more precisely capture structural disadvantage in education and employment, which are considered among the most impactful domains.^45^ The SREI’s added value lies within its analytic approach, which is not only conceptually aligned with the interconnectedness of structural racism across institutional domains, but also empirically supported as more predictive of area-level variation in life expectancy and disease prevalence than other indices.^15^

Eliminating structural racism and its harmful downstream effects on cardiovascular health requires multisector, place-based approaches to dismantle racism across interconnected social systems that limit equitable socioeconomic opportunities and contribute to racialized inequities in health.^7,27,46,47,48^ This approach includes contemporary policies and practices that are purportedly colorblind, such as the absence of any language that could be construed as considering race, but that have racist consequences.^7,8^ We found that each domain of structural racism was associated with greater neighborhood prevalence of cardiovascular risk factors and diseases. Therefore, targeted interventions must address each component that reproduces and maintains structural racism. The income and poverty domain and the education domain had the strongest association with neighborhood cardiovascular health compared with all other domains. The income and poverty domain measures neighborhood income, poverty, and the proportion of the population that receives public assistance. Structural racism contributes to the patterning of neighborhoods by perceived social value (eg, race and socioeconomic status) and deprives certain neighborhoods of resources while advantaging others.^10,49^ This system results in neighborhoods with a large composition of racially minoritized residents to experience disenfranchisement, have inadequate access to social services and economic resources, and have a greater exposure to socioenvironmental stressors, all related to health and lifespan.^10,50,51^ Interventions targeting the income and poverty domain should aim to eliminate poverty, provide opportunities for upward financial mobility, and increase political capital among residents in these neighborhoods. Policy interventions for the education domain would have to address inequities in public school funding. For example, public schools that educate a large proportion of racially minoritized students are more likely to spend less per pupil and have fewer resources due to a lower community tax base because of low property values.^10,52^ This environment imperils neighborhood educational attainment and reduces the likelihood of socioeconomic mobility, thus hindering access to health-promoting resources.^10^ Eliminating these sources of structural racism would improve health outcomes not only for racially minoritized groups but for all people.^53^ Though structural racism disproportionately impacts neighborhoods with a higher composition of racially minoritized residents, the present study showed that its effects are seen across all racial and ethnics groups living in areas with high levels of structural racism, including areas with a larger proportion of non-Hispanic White residents, such as those who live in the Appalachian region of the US, where the neighborhood disadvantage in these areas have a similar impact on health as structural racism. Place-based initiatives should reinvest in racially marginalized and underserved communities by dismantling systems that perpetuate structural racism and restructuring systems to improve conditions that affect health.

### Limitations

Several limitations must be noted. First, this study consisted of cross-sectional analyses; therefore, we cannot draw causal inferences. Second, the analyses used aggregate data at the census tract–level and are thus subject to ecological fallacy, and the findings might not apply at the individual level. Further, because the study uses aggregate outcomes, we were not able to examine racial and ethnic differences in prevalence. Third, although we adjusted for neighborhood demographic characteristics, it is possible that there are other important covariates that were not captured by the study datasets. However, results were consistent after performing geospatial- and covariate-based matching analyses to address the potential for unobserved confounders. Fourth, cardiovascular risk factor and disease prevalences were based on self-report and therefore subject to social desirability and recall bias.^45,46^ Fifth, all SREI variables were equally weighted within each domain and all domains equally constructed; therefore, they do not account for potential differing impact on cardiovascular risk factors and diseases.^15^ Lastly, there are likely other relevant indicators that were not included in the structural racism measure, such as zoning laws, lack of political representation, voting accessibility, and gentrification.

## Conclusions

This cross-sectional study found that across US neighborhoods, greater structural racism was associated with higher prevalence rates of cardiovascular risk factors and cardiovascular diseases. Our results further indicated that greater exposure to structural racism was negatively associated with cardiovascular health independent of neighborhood-level racial and ethnic composition. Future research is needed to confirm our findings using individual-level data to inform policy development centered on mitigating inequities in neighborhood cardiovascular health.

## Notes

Supplement 1. **eTable 1.** Detailed description of the Structural Racism Effect Index **eTable 2.** CDC PLACES definition for health outcomes and health status **eTable 3.** Structural racism by cardiovascular risk factor and cardiovascular disease prevalence **eTable 4.** Unadjusted prevalence rate ratios for cardiovascular risk factors and cardiovascular diseases by quintile of structural racism **eFigure 1.** Adjusted prevalence rate ratios for cardiovascular behavioral risk factors by structural racism quintile **eTable 5.** Adjusted prevalence rate ratios for cardiovascular risk factors and cardiovascular diseases by domains of structural racism **eTable 6.** Adjusted prevalence rate ratios for cardiovascular behavioral risk factors and cardiovascular disease by domains of structural racism **eTable 7.** Adjusted prevalence rate ratios for cardiovascular risk factors and cardiovascular diseases by neighborhood structural racism and proportion Asian American Population **eTable 8.** Adjusted prevalence rate ratios for cardiovascular risk factors and cardiovascular diseases by neighborhood structural racism and proportion Black population **eTable 9.** Adjusted prevalence rate ratios for cardiovascular risk factors and cardiovascular diseases by neighborhood structural racism and proportion Hispanic or Latino population **eTable 10.** Adjusted prevalence rate ratios for cardiovascular risk factors and cardiovascular diseases by neighborhood structural racism and proportion White population **eFigure 2.** Adjusted prevalence rate ratios for cardiovascular risk factor and cardiovascular diseases by structural racism adjusting for current cigarette smoking **eFigure 3.** Adjusted prevalence rate ratios for cardiovascular clinical risk factors and cardiovascular diseases by structural, not adjusting for percentage of Non-Hispanic White population **eFigure 4.** Adjusted prevalence rate ratios for cardiovascular behavioral risk factors by structural racism, not adjusting for percentage of Non-Hispanic White population

Supplement 2.
Data Sharing Statement

## References

[1] Martin SS, Aday AW, Almarzooq ZI, ; American Heart Association Council on Epidemiology and Prevention Statistics Committee and Stroke Statistics Subcommittee. 2024 Heart Disease and Stroke Statistics: A Report of US and Global Data From the American Heart Association. Circulation. 2024;149(8):e347-e913. doi:10.1161/CIR.0000000000001209 38264914 PMC12146881

[2] Powell-Wiley TM, Baumer Y, Baah FO, . Social determinants of cardiovascular disease. Circ Res. 2022;130(5):782-799. doi:10.1161/CIRCRESAHA.121.319811 35239404 PMC8893132

[3] Kyalwazi AN, Loccoh EC, Brewer LC, . Disparities in cardiovascular mortality between Black and White adults in the United States, 1999 to 2019. Circulation. 2022;146(3):211-228. doi:10.1161/CIRCULATIONAHA.122.060199 35861764 PMC9310198

[4] Williams DR, Lawrence JA, Davis BA. Racism and health: evidence and needed research. Annu Rev Public Health. 2019;40(1):105-125. doi:10.1146/annurev-publhealth-040218-043750 30601726 PMC6532402

[5] Javed Z, Haisum Maqsood M, Yahya T, . Race, racism, and cardiovascular health: applying a social determinants of health framework to racial/ethnic disparities in cardiovascular disease. Circ Cardiovasc Qual Outcomes. 2022;15(1):e007917. doi:10.1161/CIRCOUTCOMES.121.007917 35041484

[6] Kershaw KN, Osypuk TL, Do DP, De Chavez PJ, Diez Roux AV. Neighborhood-level racial/ethnic residential segregation and incident cardiovascular disease: the multi-ethnic study of atherosclerosis. Circulation. 2015;131(2):141-148. doi:10.1161/CIRCULATIONAHA.114.011345 25447044 PMC4293329

[7] Bailey ZD, Krieger N, Agénor M, Graves J, Linos N, Bassett MT. Structural racism and health inequities in the USA: evidence and interventions. Lancet. 2017;389(10077):1453-1463. doi:10.1016/S0140-6736(17)30569-X 28402827

[8] Agénor M, Perkins C, Stamoulis C, . Developing a database of structural racism-related state laws for health equity research and practice in the United States. Public Health Rep. 2021;136(4):428-440. doi:10.1177/0033354920984168 33617383 PMC8203034

[9] Diez Roux AV, Mair C. Neighborhoods and health. Ann N Y Acad Sci. 2010;1186(1):125-145. doi:10.1111/j.1749-6632.2009.05333.x 20201871

[10] Gilbert KL, Ransome Y, Dean LT, DeCaille J, Kawachi I. Social capital, Black social mobility, and health disparities. Annu Rev Public Health. 2022;43(1):173-191. doi:10.1146/annurev-publhealth-052020-112623 34990220 PMC10195010

[11] Ford CL, Griffith DM, Bruce MA, Gilbert KL, eds. Racism: Science & Tools for the Public Health Professional. American Public Health Association; 2019. doi:10.2105/9780875533049

[12] Adkins-Jackson PB, Chantarat T, Bailey ZD, Ponce NA. Measuring structural racism: a guide for epidemiologists and other health researchers. Am J Epidemiol. 2022;191(4):539-547. doi:10.1093/aje/kwab239 34564723 PMC9077112

[13] Bailey ZD, Feldman JM, Bassett MT. How structural racism works - racist policies as a root cause of U.S. racial health inequities. N Engl J Med. 2021;384(8):768-773. doi:10.1056/NEJMms2025396 33326717 PMC11393777

[14] Ghaferi AA, Schwartz TA, Pawlik TM. STROBE reporting guidelines for observational studies. JAMA Surg. 2021;156(6):577-578. doi:10.1001/jamasurg.2021.0528 33825815

[15] Dyer Z, Alcusky MJ, Galea S, Ash A. Measuring the enduring imprint of structural racism on American neighborhoods. Health Aff (Millwood). 2023;42(10):1374-1382. doi:10.1377/hlthaff.2023.00659 37782878 PMC10804769

[16] Greenlund KJ, Lu H, Wang Y, . PLACES: local data for better health. Prev Chronic Dis. 2022;19:E31. doi:10.5888/pcd19.210459 35709356 PMC9258452

[17] Zhang X, Holt JB, Yun S, Lu H, Greenlund KJ, Croft JB. Validation of multilevel regression and poststratification methodology for small area estimation of health indicators from the behavioral risk factor surveillance system. Am J Epidemiol. 2015;182(2):127-137. doi:10.1093/aje/kwv002 25957312 PMC4554328

[18] Wang Y, Holt JB, Zhang X, . Comparison of methods for estimating prevalence of chronic diseases and health behaviors for small geographic areas: Boston Validation Study, 2013. Prev Chronic Dis. 2017;14:E99. doi:10.5888/pcd14.170281 29049020 PMC5652237

[19] Williams DR. Race and health: basic questions, emerging directions. Ann Epidemiol. 1997;7(5):322-333. doi:10.1016/S1047-2797(97)00051-3 9250627

[20] Jones CP. Levels of racism: a theoretic framework and a gardener’s tale. Am J Public Health. 2000;90(8):1212-1215. doi:10.2105/AJPH.90.8.1212 10936998 PMC1446334

[21] Franklin AJ, Boyd-Franklin N, Kelly S. Racism and invisibility. J Emotional Abuse. 2006;6(2-3):9-30. doi:10.1300/J135v06n02_02

[22] Mohottige D, Davenport CA, Bhavsar N, . Residential structural racism and prevalence of chronic health conditions. JAMA Netw Open. 2023;6(12):e2348914. doi:10.1001/jamanetworkopen.2023.48914 38127347 PMC10739116

[23] Liu M, Patel VR, Salas RN, . Neighborhood environmental burden and cardiovascular health in the US. JAMA Cardiol. 2024;9(2):153-163. doi:10.1001/jamacardio.2023.4680 37955891 PMC10644252

[24] Needham BL, Ali T, Allgood KL, Ro A, Hirschtick JL, Fleischer NL. Institutional racism and health: a framework for conceptualization, measurement, and analysis. J Racial Ethn Health Disparities. 2023;10(4):1997-2019. doi:10.1007/s40615-022-01381-9 35994173 PMC9395863

[25] Schwartz GL, Wang G, Kershaw KN, McGowan C, Kim MH, Hamad R. The long shadow of residential racial segregation: associations between childhood residential segregation trajectories and young adult health among Black US Americans. Health Place. 2022;77:102904. doi:10.1016/j.healthplace.2022.102904 36063651 PMC10166594

[26] Kong AY, Herbert L, Feldman JM, Trangenstein PJ, Fakunle DO, Lee JGL. Tobacco and alcohol retailer availability and neighborhood racialized, economic, and racialized economic segregation in North Carolina. J Racial Ethn Health Disparities. 2023;10(6):2861-2871. doi:10.1007/s40615-022-01463-8 36469288 PMC11809087

[27] Albert MA, Churchwell K, Desai N, ; American Heart Association Advocacy Coordinating Committee. Addressing structural racism through public policy advocacy: a policy statement from the American Heart Association. Circulation. 2024;149(6):e312-e329. doi:10.1161/CIR.0000000000001203 38226471

[28] Lee JGL, Henriksen L, Rose SW, Moreland-Russell S, Ribisl KM. A systematic review of neighborhood disparities in point-of-sale tobacco marketing. Am J Public Health. 2015;105(9):e8-e18. doi:10.2105/AJPH.2015.302777 26180986 PMC4529779

[29] Stuart EA. Matching methods for causal inference: a review and a look forward. Stat Sci. 2010;25(1):1-21. doi:10.1214/09-STS313 20871802 PMC2943670

[30] Kershaw KN, Magnani JW, Diez Roux AV, ; Council on Quality of Care and Outcomes Research; Council on Epidemiology and Prevention; Council on Clinical Cardiology; Council on Hypertension; Council on Cardiovascular and Stroke Nursing; Council on Peripheral Vascular Disease; and Council on the Kidney in Cardiovascular Disease. Neighborhoods and cardiovascular health: a scientific statement from the American Heart Association. Circ Cardiovasc Qual Outcomes. 2024;17(1):e000124. doi:10.1161/HCQ.0000000000000124 38073532

[31] Pascoe EA, Smart Richman L. Perceived discrimination and health: a meta-analytic review. Psychol Bull. 2009;135(4):531-554. doi:10.1037/a0016059 19586161 PMC2747726

[32] Lawrence WR, Jones GS, Johnson JA, . Discrimination experiences and all-cause and cardiovascular mortality: multi-ethnic study of atherosclerosis. Circ Cardiovasc Qual Outcomes. 2023;16(4):e009697. doi:10.1161/CIRCOUTCOMES.122.009697 37017086 PMC10106108

[33] Burroughs Peña MS, Mbassa RS, Slopen NB, Williams DR, Buring JE, Albert MA. Cumulative psychosocial stress and ideal cardiovascular health in older women. Circulation. 2019;139(17):2012-2021. doi:10.1161/CIRCULATIONAHA.118.033915 30813768 PMC6478505

[34] Xiao Q, Heiss G, Kucharska-Newton A, Bey G, Love SM, Whitsel EA. Life-course neighborhood socioeconomic status and cardiovascular events in Black and White adults in the Atherosclerosis Risk in Communities Study. Am J Epidemiol. 2022;191(8):1470-1484. doi:10.1093/aje/kwac070 35419583 PMC9989355

[35] Hines AL, Albert MA, Blair JP, . Neighborhood factors, individual stressors, and cardiovascular health among Black and White adults in the US: the Reasons for Geographic and Racial Differences in Stroke (REGARDS) Study. JAMA Netw Open. 2023;6(9):e2336207. doi:10.1001/jamanetworkopen.2023.36207 37773494 PMC10543067

[36] Malla G, Long DL, Cherrington A, . Neighborhood disadvantage and risk of heart failure: the Reasons for Geographic and Racial Differences in Stroke (REGARDS) study. Circ Cardiovasc Qual Outcomes. 2024;17(3):e009867. doi:10.1161/CIRCOUTCOMES.123.009867 38328917 PMC10950536

[37] Geronimus AT, Hicken MT, Pearson JA, Seashols SJ, Brown KL, Cruz TD. Do US Black women experience stress-related accelerated biological aging?: a novel theory and first population-based test of Black-White differences in telomere length. Hum Nat. 2010;21(1):19-38. doi:10.1007/s12110-010-9078-0 20436780 PMC2861506

[38] Sangaramoorthy M, Samayoa C, Inamdar PP, . Association between neighborhood stressors and allostatic load in breast cancer survivors: the Pathways Study. Am J Epidemiol. 2025;194(5):1264-1274. doi:10.1093/aje/kwae134 38896063 PMC12055468

[39] Williams DR, Mohammed SA. Racism and Health I: pathways and scientific evidence. Am Behav Sci. 2013;57(8):1152-1173. doi:10.1177/0002764213487340 24347666 PMC3863357

[40] Suglia SF, Campo RA, Brown AGM, . Social determinants of cardiovascular health: early life adversity as a contributor to disparities in cardiovascular diseases. J Pediatr. 2020;219:267-273. doi:10.1016/j.jpeds.2019.12.063 32111376 PMC7883398

[41] Shonkoff JP, Slopen N, Williams DR. Early childhood adversity, toxic stress, and the impacts of racism on the foundations of health. Annu Rev Public Health. 2021;42(1):115-134. doi:10.1146/annurev-publhealth-090419-101940 33497247

[42] Makuvire TT, Lopez JL, Latif Z, . The application of neighborhood area deprivation index to improve health equity across the spectrum of heart failure: a review. Heart Fail Rev. 2025;30(3):589-604. doi:10.1007/s10741-025-10492-4 40158031

[43] Trinidad S, Brokamp C, Mor Huertas A, . Use of area-based socioeconomic deprivation indices: a scoping review and qualitative analysis. Health Aff (Millwood). 2022;41(12):1804-1811. doi:10.1377/hlthaff.2022.00482 36469826

[44] Jahn JL. Invited commentary: comparing approaches to measuring structural racism. Am J Epidemiol. 2022;191(4):548-551. doi:10.1093/aje/kwab261 34718384 PMC9630390

[45] Lou S, Giorgi S, Liu T, Eichstaedt JC, Curtis B. Measuring disadvantage: a systematic comparison of United States small-area disadvantage indices. Health Place. 2023;80:102997. doi:10.1016/j.healthplace.2023.102997 36867991 PMC10038931

[46] Zubizarreta D, Beccia AL, Chen JT, Jahn JL, Austin SB, Agénor M. Structural racism-related state laws and healthcare access among Black, Latine, and White U.S. adults. J Racial Ethn Health Disparities. 2025;12(3):1432-1445. doi:10.1007/s40615-024-01976-4 38546945 PMC11542902

[47] Ibrahim R, Sainbayar E, Pham HN, . Social vulnerability index and cardiovascular disease care continuum: a scoping review. JACC Adv. 2024;3(7):100858. doi:10.1016/j.jacadv.2024.100858 39130018 PMC11312302

[48] Churchwell K, Elkind MSV, Benjamin RM, ; American Heart Association. Call to action: structural racism as a fundamental driver of health disparities: a presidential advisory from the American Heart Association. Circulation. 2020;142(24):e454-e468. doi:10.1161/CIR.0000000000000936 33170755

[49] Lawrence WR, Freedman ND, McGee-Avila JK, . Contemporary neighborhood redlining and racial mortgage lending bias and disparities in prostate cancer survival. Cancer. 2025;131(8):e35850. doi:10.1002/cncr.35850 40233138 PMC12001745

[50] Phelan JC, Link BG. Is racism a fundamental cause of inequalities in health? Annu Rev Sociol. 2015;41(1):311-330. doi:10.1146/annurev-soc-073014-112305

[51] Brown TH, Lee HE, Hicken MT, Bonilla-Silva E, Homan P. Conceptualizing and measuring systemic racism. Annu Rev Public Health. 2025;46(1):69-90. doi:10.1146/annurev-publhealth-060222-032022 39809467 PMC13288184

[52] Johnson O. Is concentrated advantage the cause? The relative contributions of neighborhood advantage and disadvantage to educational inequality. Urban Rev. 2013;45(5):561-585. doi:10.1007/s11256-013-0242-9

[53] Jones CP. 11. Action and Allegories. In: Racism: Science & Tools for the Public Health Professional. American Public Health Association; 2019, doi:10.2105/9780875533049ch11

