# Supplementary material for: Manifestations of Structural Racism and Inequities in Cardiovascular Health Across US Neighborhoods
Source: JAMA Health Forum. 2025 Oct 31;6(10):e253864. doi: 10.1001/jamahealthforum.2025.3864 (PMC12579350; doi:10.1001/jamahealthforum.2025.3864)
Supplement: Supplement 1. — eTable 1. Detailed description of the Structural Racism Effect Index eTable 2. CDC PLACES definition for health outcomes and health status eTable 3. Structural racism by cardiovascular risk factor and cardiovascular disease prevalence eTable 4. Unadjusted prevalence rate ratios for cardiovascular risk factors and cardiovascular diseases by quintile of structural racism eFigure 1. Adjusted prevalence rate ratios for cardiovascular behavioral risk factors by structural racism quintile eTable 5. Adjusted prevalence rate ratios for cardiovascular risk factors and cardiovascular diseases by domains of structural racism eTable 6. Adjusted prevalence rate ratios for cardiovascular behavioral risk factors and cardiovascular disease by domains of structural racism eTable 7. Adjusted prevalence rate ratios for cardiovascular risk factors and cardiovascular diseases by neighborhood structural racism and proportion Asian American Population eTable 8. Adjusted prevalence rate ratios for cardiovascular risk factors and cardiovascular diseases by neighborhood structural racism and proportion Black population eTable 9. Adjusted prevalence rate ratios for cardiovascular risk factors and cardiovascular diseases by neighborhood structural racism and proportion Hispanic or Latino population eTable 10. Adjusted prevalence rate ratios for cardiovascular risk factors and cardiovascular diseases by neighborhood structural racism and proportion White population eFigure 2. Adjusted prevalence rate ratios for cardiovascular risk factor and cardiovascular diseases by structural racism adjusting for current cigarette smoking eFigure 3. Adjusted prevalence rate ratios for cardiovascular clinical risk factors and cardiovascular diseases by structural, not adjusting for percentage of Non-Hispanic White population eFigure 4. Adjusted prevalence rate ratios for cardiovascular behavioral risk factors by structural racism, not adjusting for percentage of Non-Hispanic White population [file jamahealthforum-e253864-s001.pdf]

Supplementary Online Content

Lawrence WR, Hong HG, Williams F, et al. Manifestations of structural racism and inequities in cardiovascular health across US neighborhoods. *JAMA Health Forum*. Published October 31, 2025. doi: 10.1001/jamahealthforum.2025.3864

eTable 1. Detailed description of the Structural Racism Effect Index
eTable 2. CDC PLACES definition for health outcomes and health status
eTable 3. Structural racism by cardiovascular risk factor and cardiovascular disease prevalence
eTable 4. Unadjusted prevalence rate ratios for cardiovascular risk factors and cardiovascular diseases by quintile of structural racism
eFigure 1. Adjusted prevalence rate ratios for cardiovascular behavioral risk factors by structural racism quintile
eTable 5. Adjusted prevalence rate ratios for cardiovascular risk factors and cardiovascular diseases by domains of structural racism
eTable 6. Adjusted prevalence rate ratios for cardiovascular behavioral risk factors and cardiovascular disease by domains of structural racism
eTable 7. Adjusted prevalence rate ratios for cardiovascular risk factors and cardiovascular diseases by neighborhood structural racism and proportion Asian American Population
eTable 8. Adjusted prevalence rate ratios for cardiovascular risk factors and cardiovascular diseases by neighborhood structural racism and proportion Black population
eTable 9. Adjusted prevalence rate ratios for cardiovascular risk factors and cardiovascular diseases by neighborhood structural racism and proportion Hispanic or Latino population
eTable 10. Adjusted prevalence rate ratios for cardiovascular risk factors and cardiovascular diseases by neighborhood structural racism and proportion White population
eFigure 2. Adjusted prevalence rate ratios for cardiovascular risk factor and cardiovascular diseases by structural racism adjusting for current cigarette smoking
eFigure 3. Adjusted prevalence rate ratios for cardiovascular clinical risk factors and cardiovascular diseases by structural, not adjusting for percentage of Non-Hispanic White population
eFigure 4. Adjusted prevalence rate ratios for cardiovascular behavioral risk factors by structural racism, not adjusting for percentage of Non-Hispanic White population

This supplemental material has been provided by the authors to give readers additional information about their work.

© 2025 Lawrence WR et al. JAMA Health Forum.

eTable 1. Detailed Description of the Structural Racism Effect Index

	Domains	Years [a]	Indicators	Indicator Descriptions
**1**	Built Environment	2015-2019	Building vacancy rate	Mean vacancy rate
		2015-2019	Mobile homes	Proportion of mobile homes
		2015-2019	No internet access	Proportion of households with internet access
		2018	Cancer risk	Total air toxins and cancer risk due to air pollutant exposure (Total Cancer Risk per million)
		2021	Low food access for SNAP recipients	Share of tract housing units receiving SNAP benefits count beyond 1 mile from supermarket
**2**	Criminal Justice	2016	Pretrial jail rate	Total jail pretrial rate
		2016	Total jail rate	Total jail population rate
		2019	Law enforcement personnel per capita	Total law enforcement employees per population
**3**	Education	2015-2019	No bachelor's degree	Proportion of population aged ≥ 25 years without a bachelor's degree
		2015-2019	Highest degree high school diploma	Proportion of population aged ≥ 25 years with highest educational attainment is a high school degree
		2019	Per pupil spending	School system per pupil spending
**4**	Employment	2015-2019	Unemployed	Proportion of civilian labor force unemployed
		2015-2019	White-collar occupations	Proportion of population with management, business, science, and arts occupations
		2019	Retail job availability	Retail jobs for every tract in the US divided by its distance from the centroid squared
**5**	Housing	2016	Eviction rate	Ratio of the number of renter-occupied households in an area that received an eviction judgement in which renters were ordered to leave
		2010	Foreclosure risk	Estimated percent of mortgages to start foreclosure process or be seriously delinquent in past 2 years
		2015-2019	Units without telephone	Proportion of owner occupied no telephone service available, and renter occupied no telephone service available
		2015-2019	Units without plumbing	Proportion of owner occupied lacking complete plumbing facilities and renter occupied lacking complete plumbing facilities
		2015-2019	Crowding	Proportion of owner and renter occupied occupants per room
		2015-2019	Group quarters	Proportion of Population in group quarters
**6**	Income and Poverty	2015-2019	Below federal poverty level	Proportion of income in the past 12 months below poverty level
		2015-2019	Below 200% poverty level	Percent of population with income below 200% of federal poverty level

© 2025 Lawrence WR et al. JAMA Health Forum.

		2015-2019	Public assistance	Proportion of people with public assistance income in the past 12 months for households
		2015-2019	Family income	Median family income in the past 12 months (in 2019 inflation-adjusted dollars)
		2015-2019	Per capita income	Per capita income in the past 12 months (in 2019 inflation-adjusted dollars)
		2018	Supplemental poverty measure	Extends the official poverty measure by considering government benefits and necessary expenses (e.g., taxes).
**7**	Social Cohesion	2012	Residential Segregation	Location Quotient: Black or African American Alone, Hispanic, and Asian or Pacific Islander<br>Index of Concentration at Extremes (ICE): Black or African American Alone/White Alone, Asian or Pacific Islander Alone/ White Alone, Hispanic-any race Alone/White Alone
		2015-2019	Changed address in the past year	Same house 1 year ago
		2015-2019	Single-parent household	Single-parent household with own children under 18 years!
		2015-2019	Income gap	Ratio of households making less than $10,000 to those making more than $50,000.
**8**	Transportation	2019	Transportation cost burden	Transportation cost burden, median income family
		2015-2019	Carpooled to work	Proportion of population carpooled to work by car, truck, or van
		2015-2019	No access to motor vehicle	Proportion of population with no owner-occupied vehicle, no renter occupied vehicle available
		2015-2019	Took public transit to work	Proportion of population that uses public transportation to work
		2015-2019	Walked to work	Proportion of population that walked to work
		2015-2019	Biked to work	Proportion of population that bicycle to work
**9**	Wealth	2015-2019	Aggregate home value	Aggregate home value in dollars
		2015-2019	Median real estate taxes paid	Median real estate taxes paid in dollars
		2015-2019	Median home value	Median home value in dollars
		2015-2019	Median gross rent	Median gross rent in dollars
		2015-2019	Median monthly mortgage	Housing units with a mortgage, median selected monthly owner costs in dollars.
		2015-2019	Owner-occupied homes	Proportion of owner-occupied homes

[a] The most recent data available at the time of Structural Racism Effect Index development was used.

© 2025 Lawrence WR et al. JAMA Health Forum.

eTable 2: CDC Places Definition for Health Outcomes and Health Status

Current Cigarette Smoking	Among adults aged ≥ 18 years who report having smoked ≥ 100 cigarettes in their lifetime and currently smoke every day or some days
No leisure-time physical activity among adults	Among adults aged ≥ 18 years who report ‘No’ to the question: “During the past month, other than your regular job, did you participate in any physical activities or exercises such as running, calisthenics, golf, gardening, or walking for exercise?”
High Blood Pressure	Among adults aged ≥ 18 years who report ever having been told by a doctor, nurse, or other health professional that they have high blood pressure.
High Cholesterol	Among adults aged ≥ 18 years who report having ever been screened for high cholesterol and told by a doctor, nurse, or other health professional that they had high cholesterol.
Obesity	Among adults aged ≥18 years who have a body mass index ≥30.0 kg/m^2 calculated from self-reported weight and height.
Diabetes	Among adults aged ≥ 18 years who report being told by a doctor or other health professional that they have diabetes (other than diabetes during pregnancy for female respondents).
Coronary Heart Disease	Among adults aged ≥ 18 years who report ever having been told by a doctor, nurse, or other health professional that they had angina or coronary heart disease.
Stroke	Among adults aged ≥ 18 years who report ever having been told by a doctor, nurse, or other health professional that they have had a stroke.

© 2025 Lawrence WR et al. JAMA Health Forum.

eTable 3. Structural Racism by Cardiovascular Risk Factor and Cardiovascular Disease Prevalence

Characteristics	All neighborhoods (n= 71,915)	First (lowest) (n= 14,383)	Second (14,383)	Third (14,384)	Fourth (14,383)	Fifth (highest) (14,382)
Cardiovascular Behavioral Risk Factors						
Current cigarette smoking, % mean (SD)	18.3 (5.8)	11.7 (2.7)	15.5 (3.0)	18.1 (3.4)	20.7 (3.7)	25.3 (4.7)
No leisure-time physical activity, % mean (SD)	25.4 (7.6)	17.2 (3.7)	21.4 (3.9)	24.6 (3.9)	28.5 (4.2)	35.6 (5.5)
Cardiovascular Clinical Risk Factors						
High Blood Pressure, % mean (SD)	32.4 (7.2)	27.1 (5.3)	29.8 (5.3)	31.8 (5.5)	34.1 (6.1)	39.0 (7.5)
High Cholesterol, % mean (SD)	33.5 (4.7)	31.9 (4.5)	32.6 (4.3)	33.5 (4.6)	34.3 (4.8)	35.1 (4.5)
Obesity, % mean (SD)	32.1 (6.9)	24.7 (4.3)	29.1 (4.3)	31.8 (4.2)	34.7 (4.0)	40.3 (5.2)
Diabetes, % mean (SD)	11.2 (3.7)	8.0 (1.8)	9.4 (2.0)	10.6 (2.2)	12.2 (2.5)	15.8 (3.9)
Cardiovascular Diseases						
Coronary Heart Disease, % mean (SD)	6.7 (2.2)	5.0 (1.5)	5.9 (1.7)	6.6 (1.8)	7.3 (2.0)	8.4 (2.1)
Stroke, % mean (SD)	3.4 (1.2)	2.4 (0.6)	2.9 (0.7)	3.3 (0.8)	3.7 (0.9)	4.9 (1.4)

Abbreviations: SD, Standard Deviation

Note: Higher quintiles indicate greater negative effects of structural racism.

© 2025 Lawrence WR et al. JAMA Health Forum.

eTable 4. Unadjusted Prevalence Rate Ratios for Cardiovascular Risk Factors and Cardiovascular Diseases by Quintile of Structural Racism

	Prevalence Risk Ratio (95% CI) [a]				
Characteristics	Quintile 1 (n= 14,383)	Quintile 2 (14,383)	Quintile 3 (14,384)	Quintile 4 (14,383)	Quintile 5 (14,382)
Cardiovascular Behavioral Risk Factors					
Current Cigarette Smoking	Reference	1.28 (1.27-1.28)	1.49 (1.48-1.49)	1.73 (1.72-1.74)	2.13 (2.12-2.13)
No Leisure Time Physical Activity	Reference	1.25 (1.25-1.26)	1.45 (1.45-1.46)	1.70 (1.69-1.70)	2.10 (2.10-2.11)
Cardiovascular Clinical Risk Factors					
High Blood Pressure	Reference	1.05 (1.05-1.06)	1.10 (1.09-1.10)	1.15 (1.15-1.16)	1.29 (1.29-1.30)
High Cholesterol	Reference	0.99 (0.99-0.99)	0.99 (0.98-0.99)	0.99 (0.99-1.00)	1.02 (1.01-1.02)
Obesity	Reference	1.12 (1.12-1.12)	1.21 (1.21-1.21)	1.33 (1.32-1.33)	1.53 (1.53-1.54)
Diabetes	Reference	1.16 (1.15-1.17)	1.29 (1.29-1.30)	1.48 (1.47-1.49)	1.88 (1.87-1.89)
Cardiovascular Diseases					
Coronary Heart Disease	Reference	1.09 (1.08-1.09)	1.17 (1.16-1.17)	1.26 (1.26-1.27)	1.46 (1.45-1.47)
Stroke	Reference	1.16 (1.15-1.17)	1.30 (1.29-1.30)	1.47 (1.46-1.48)	1.89 (1.88-1.91)

[a] Linear mixed models weighted for population size and clustered at the county level.
Whiskers represent 95% CI.
Q1=reference group.
Note: Higher quintiles indicate greater negative effects of structural racism.
Abbreviations: 95%CI, 95% confidence interval

© 2025 Lawrence WR et al. JAMA Health Forum.

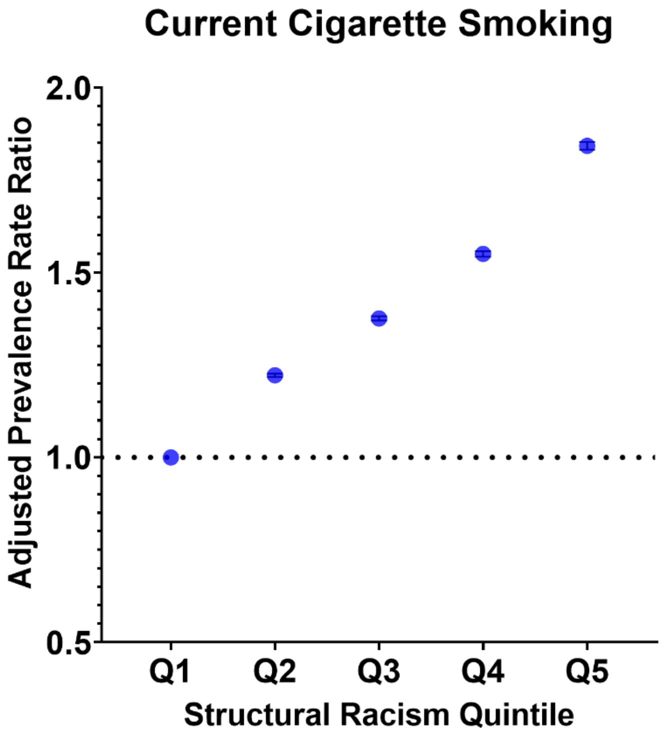

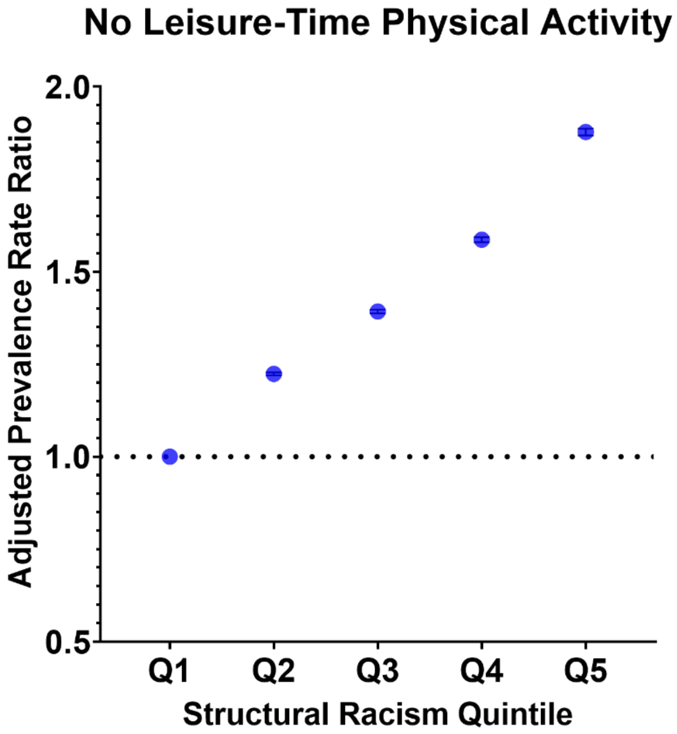

eFigure 1. Adjusted Prevalence Rate Ratios for Cardiovascular Behavioral Risk Factors by Structural Racism Quintile.

Multilevel linear mixed models weighted for population size and clustered at the county level, adjusting for age (median age), sex (percentage female), percentage of non-Hispanic White population, metropolitan status, region (Midwest, Northeast, Pacific, South, or West,), number of cardiovascular disease physicians per 100,000 population, number of primary care physicians per 100 000 population, percentage of adults having received a routine checkup in the last year, percent uninsured adults. Q1=reference group.
Abbreviations Q=quintile
Note: Higher quintiles indicate greater negative effects of structural racism. Whiskers represent 95% CI.

© 2025 Lawrence WR et al. JAMA Health Forum.

eTable 5. Adjusted Prevalence Rate Ratios for Cardiovascular Risk Factors and Cardiovascular Diseases by Domains of Structural Racism [a]

	High Blood Pressure APRR (95% CI)	High Cholesterol APRR (95% CI)	Obesity APRR (95% CI)	Diabetes APRR (95% CI)	Coronary Heart Disease APRR (95% CI)	Stroke APRR (95% CI)
Built Environment						
Quintile 1	Reference	Reference	Reference	Reference	Reference	Reference
Quintile 2	1.05 (1.05-1.05)	1.02 (1.02-1.03)	1.04 (1.03-1.04)	1.08 (1.08-1.09)	1.11 (1.10-1.11)	1.12 (1.11-1.12)
Quintile 3	1.09 (1.09-1.10)	1.05 (1.04-1.05)	1.07 (1.07-1.08)	1.17 (1.16-1.17)	1.20 (1.19-1.21)	1.21 (1.21-1.22)
Quintile 4	1.16 (1.15-1.16)	1.08 (1.08-1.08)	1.14 (1.13-1.14)	1.30 (1.29-1.30)	1.32 (1.32-1.33)	1.36 (1.35-1.37)
Quintile 5	1.21 (1.20-1.21)	1.10 (1.10-1.11)	1.20 (1.20-1.21)	1.42 (1.41-1.43)	1.44 (1.44-1.45)	1.52 (1.51-1.53)
Criminal Justice						
Quintile 1	Reference	Reference	Reference	Reference	Reference	Reference
Quintile 2	1.00 (1.00-1.01)	1.00 (1.00-1.00)	1.01 (1.01-1.02)	1.00 (0.99-1.01)	0.99 (0.99-1.00)	1.00 (1.00-1.01)
Quintile 3	1.00 (1.00-1.01)	1.00 (0.99-1.00)	1.01 (1.01-1.01)	0.99 (0.98-1.00)	0.99 (0.98-1.00)	1.00 (0.99-1.01)
Quintile 4	1.01 (1.01-1.02)	1.00 (0.99-1.00)	1.02 (1.02-1.03)	1.01 (1.00-1.02)	1.01 (1.00-1.01)	1.02 (1.01-1.03)
Quintile 5	1.01 (1.00-1.01)	0.99 (0.99 -1.00)	1.03 (1.02-1.04)	1.02 (1.01-1.03)	1.00 (0.99-1.01)	1.03 (1.02-1.04)
Education						
Quintile 1	Reference	Reference	Reference	Reference	Reference	Reference
Quintile 2	1.10 (1.10-1.10)	1.06 (1.06-1.06)	1.10 (1.09-1.10)	1.19 (1.18-1.19)	1.18 (1.18-1.19)	1.21 (1.20-1.21)
Quintile 3	1.18 (1.17-1.18)	1.10 (1.10-1.10)	1.16 (1.16-1.17)	1.33 (1.33-1.34)	1.33 (1.33-1.34)	1.38 (1.37-1.39)
Quintile 4	1.25 (1.25-1.25)	1.15 (1.14-1.15)	1.23 (1.22-1.23)	1.50 (1.49-1.50)	1.50 (1.49-1.51)	1.57 (1.56-1.58)
Quintile 5	1.36 (1.35-1.36)	1.21 (1.21-1.21)	1.32 (1.32-1.33)	1.77 (1.76-1.78)	1.76 (1.75-1.77)	1.87 (1.86-1.88)
Employment						
Quintile 1	Reference	Reference	Reference	Reference	Reference	Reference
Quintile 2	1.07 (1.07-1.08)	1.03 (1.03-1.04)	1.08 (1.08-1.09)	1.13 (1.13-1.14)	1.12 (1.12-1.13)	1.15 (1.15-1.16)
Quintile 3	1.12 (1.12-1.13)	1.06 (1.05-1.06)	1.14 (1.13-1.14)	1.22 (1.21-1.23)	1.22 (1.21-1.22)	1.26 (1.26-1.27)
Quintile 4	1.16 (1.16-1.16)	1.08 (1.07-1.08)	1.18 (1.17-1.18)	1.30 (1.30-1.31)	1.30 (1.29-1.31)	1.36 (1.35-1.37)
Quintile 5	1.21 (1.21-1.22)	1.10 (1.10-1.10)	1.24 (1.24-1.25)	1.43 (1.42-1.44)	1.42 (1.41-1.43)	1.52 (1.51-1.53)
Housing						
Quintile 1	Reference	Reference	Reference	Reference	Reference	Reference
Quintile 2	1.02 (1.02-1.03)	1.01 (1.00-1.01)	1.02 (1.02-1.02)	1.04 (1.04-1.05)	1.06 (1.06-1.07)	1.07 (1.06-1.07)
Quintile 3	1.04 (1.04-1.04)	1.01 (1.01-1.01)	1.03 (1.03-1.04)	1.07 (1.07-1.08)	1.10 (1.09-1.10)	1.11 (1.11-1.12)
Quintile 4	1.06 (1.06-1.06)	1.01 (1.01-1.02)	1.06 (1.05-1.06)	1.11 (1.10-1.11)	1.14 (1.13-1.15)	1.16 (1.16-1.17)
Quintile 5	1.08 (1.08-1.09)	1.01 (1.00-1.01)	1.10 (1.10-1.10)	1.16 (1.16-1.17)	1.20 (1.19-1.21)	1.25 (1.24-1.26)
Income and Poverty						
Quintile 1	Reference	Reference	Reference	Reference	Reference	Reference
Quintile 2	1.08 (1.08-1.09)	1.03 (1.03-1.04)	1.07 (1.07-1.07)	1.15 (1.14-1.15)	1.17 (1.16-1.17)	1.19 (1.18-1.19)
Quintile 3	1.14 (1.14-1.14)	1.06 (1.06-1.06)	1.12 (1.12-1.12)	1.27 (1.26-1.27)	1.30 (1.29-1.31)	1.33 (1.33-1.34)
Quintile 4	1.20 (1.20-1.21)	1.09 (1.09-1.10)	1.18 (1.18-1.19)	1.42 (1.41-1.42)	1.46 (1.46-1.47)	1.51 (1.51-1.52)

© 2025 Lawrence WR et al. JAMA Health Forum.

Quintile 5	1.31 (1.30-1.31)	1.14 (1.14-1.14)	1.31 (1.31-1.32)	1.70 (1.69-1.71)	1.74 (1.73-1.75)	1.85 (1.84-1.87)
Social Cohesion						
Quintile 1	Reference	Reference	Reference	Reference	Reference	Reference
Quintile 2	1.03 (1.02-1.03)	1.01 (1.01-1.01)	1.02 (1.02-1.02)	1.05 (1.04-1.05)	1.07 (1.06-1.07)	1.07 (1.07-1.08)
Quintile 3	1.05 (1.05-1.05)	1.02 (1.01-1.02)	1.04 (1.04-1.04)	1.10 (1.10-1.11)	1.13 (1.12-1.13)	1.14 (1.13-1.14)
Quintile 4	1.08 (1.08-1.09)	1.02 (1.02-1.02)	1.08 (1.08-1.09)	1.17 (1.16-1.18)	1.20 (1.19-1.20)	1.23 (1.22-1.23)
Quintile 5	1.13 (1.13-1.14)	1.01 (1.00-1.01)	1.19 (1.19-1.20)	1.27 (1.26-1.28)	1.27 (1.26-1.28)	1.37 (1.36-1.38)
Transportation						
Quintile 1	Reference	Reference	Reference	Reference	Reference	Reference
Quintile 2	1.04 (1.04-1.05)	1.05 (1.04-1.05)	1.03 (1.03-1.03)	1.07 (1.07-1.08)	1.06 (1.05-1.06)	1.06 (1.05-1.06)
Quintile 3	1.06 (1.06-1.07)	1.06 (1.06-1.06)	1.05 (1.04-1.05)	1.11 (1.10-1.11)	1.09 (1.08-1.09)	1.08 (1.08-1.09)
Quintile 4	1.08 (1.08-1.08)	1.07 (1.07-1.08)	1.06 (1.06-1.07)	1.15 (1.14-1.15)	1.12 (1.11-1.13)	1.12 (1.11-1.13)
Quintile 5	1.11 (1.10-1.11)	1.09 (1.09-1.10)	1.09 (1.08-1.09)	1.21 (1.20-1.21)	1.18 (1.17-1.19)	1.19 (1.18-1.20)
Wealth						
Quintile 1	Reference	Reference	Reference	Reference	Reference	Reference
Quintile 2	1.09 (1.08-1.09)	1.03 (1.03-1.04)	1.09 (1.09-1.09)	1.16 (1.15-1.16)	1.16 (1.15-1.17)	1.19 (1.18-1.20)
Quintile 3	1.15 (1.15-1.15)	1.06 (1.06-1.06)	1.15 (1.15-1.16)	1.28 (1.27-1.28)	1.30 (1.29-1.31)	1.35 (1.35-1.36)
Quintile 4	1.20 (1.20-1.21)	1.08 (1.08-1.08)	1.20 (1.19-1.20)	1.38 (1.38-1.39)	1.43 (1.42-1.44)	1.50 (1.49-1.51)
Quintile 5	1.26 (1.25-1.26)	1.10 (1.09-1.10)	1.26 (1.26-1.27)	1.53 (1.52-1.54)	1.60 (1.59-1.61)	1.71 (1.70-1.73)

[a] Multilevel linear mixed models weighted for population size and clustered at the county level, adjusting for age (median age), sex (percentage female), percentage of non-Hispanic White population, metropolitan status, region (Midwest, Northeast, Pacific, South, or West,), number of cardiovascular disease physicians per 100,000 population, number of primary care physicians per 100,000 population, percentage of adults having received a routine checkup in the last year, percent uninsured adults.

Note: Higher quintiles indicate greater negative effects of structural racism.

Abbreviations: APRR, Adjusted Prevalence Rate Ratio; 95%CI, 95% confidence interval.

© 2025 Lawrence WR et al. JAMA Health Forum.

eTable 6 Adjusted Prevalence Rate Ratios for Cardiovascular Behavioral Risk Factors and Diseases by Domains of Structural Racism [a]

	Current Cigarette Smoking APRR (95% CI)	No Leisure-Time Physical Activity APRR (95% CI)
Built Environment		
Quintile 1	Reference	Reference
Quintile 2	1.09 (1.09-1.09)	1.09 (1.09-1.10)
Quintile 3	1.16 (1.16-1.17)	1.18 (1.17-1.18)
Quintile 4	1.28 (1.27-1.29)	1.30 (1.29-1.30)
Quintile 5	1.43 (1.42-1.44)	1.41 (1.40-1.42)
Criminal Justice		
Quintile 1	Reference	Reference
Quintile 2	1.01 (1.01-1.02)	1.00 (0.99-1.00)
Quintile 3	1.01 (1.00-1.02)	0.99 (0.98-1.00)
Quintile 4	1.04 (1.03-1.05)	1.00 (1.00-1.01)
Quintile 5	1.05 (1.03-1.06)	1.02 (1.01-1.03)
Education		
Quintile 1	Reference	Reference
Quintile 2	1.23 (1.23-1.23)	1.21 (1.21-1.21)
Quintile 3	1.39 (1.38-1.39)	1.37 (1.37-1.38)
Quintile 4	1.54 (1.53-1.54)	1.53 (1.53-1.54)
Quintile 5	1.71 (1.70-1.71)	1.78 (1.77-1.79)
Employment		
Quintile 1	Reference	Reference
Quintile 2	1.19 (1.18-1.19)	1.17 (1.16-1.17)
Quintile 3	1.30 (1.30-1.31)	1.28 (1.27-1.28)
Quintile 4	1.39 (1.38-1.40)	1.37 (1.36-1.37)
Quintile 5	1.52 (1.51-1.53)	1.49 (1.48-1.50)
Housing		
Quintile 1	Reference	Reference
Quintile 2	1.06 (1.06-1.06)	1.06 (1.05-1.06)
Quintile 3	1.10 (1.09-1.10)	1.09 (1.09-1.10)
Quintile 4	1.13 (1.13-1.14)	1.14 (1.13-1.14)
Quintile 5	1.21 (1.21-1.22)	1.21 (1.21-1.22)
Income and Poverty		
Quintile 1	Reference	Reference
Quintile 2	1.19 (1.19-1.20)	1.19 (1.18-1.19)
Quintile 3	1.31 (1.30-1.31)	1.32 (1.32-1.33)
Quintile 4	1.44 (1.43-1.45)	1.48 (1.48-1.49)
Quintile 5	1.69 (1.69-1.70)	1.77 (1.76-1.78)
Social Cohesion		
Quintile 1	Reference	Reference
Quintile 2	1.05 (1.05-1.05)	1.06 (1.06-1.06)
Quintile 3	1.08 (1.08-1.09)	1.12 (1.11-1.12)
Quintile 4	1.13 (1.12-1.14)	1.20 (1.19-1.21)
Quintile 5	1.28 (1.27-1.28)	1.34 (1.33-1.34)
Transportation		
Quintile 1	Reference	Reference
Quintile 2	1.06 (1.05-1.06)	1.06 (1.05-1.06)
Quintile 3	1.09 (1.09-1.10)	1.09 (1.08-1.10)
Quintile 4	1.12 (1.12-1.13)	1.12 (1.12-1.13)
Quintile 5	1.17 (1.16-1.17)	1.17 (1.17-1.18)

© 2025 Lawrence WR et al. JAMA Health Forum.

Wealth		
Quintile 1	Reference	Reference
Quintile 2	1.18 (1.17-1.18)	1.19 (1.19-1.20)
Quintile 3	1.32 (1.31-1.32)	1.34 (1.33-1.34)
Quintile 4	1.43 (1.42-1.44)	1.46 (1.46-1.47)
Quintile 5	1.59 (1.58-1.60)	1.62 (1.62-1.63)

[a] Multilevel linear mixed models weighted for population size and clustered at the county level, adjusting for age (median age), sex (percentage female), percentage of non-Hispanic White population, metropolitan status, region (Midwest, Northeast, Pacific, South, or West,), number of cardiovascular disease physicians per 100,000 population, number of primary care physicians per 100,000 population, percentage of adults having received a routine checkup in the last year, percent uninsured adults.

Note: Higher quintiles indicate greater negative effects of structural racism.

Abbreviations: APRR, Adjusted Prevalence Rate Ratio; 95%CI, 95% confidence interval.

© 2025 Lawrence WR et al. JAMA Health Forum.

eTable 7. Adjusted Prevalence Rate Ratios for Cardiovascular Risk Factors and Cardiovascular Diseases by Neighborhood Structural Racism and Proportion Asian Americans Population

	Quartile of the Percentage of Asian Americans Adjusted Prevalence Risk Ratio (95%CI) [a]				
	First (lowest)	Second	Third	Fourth (Highest)	P interaction [b]
**Cardiovascular Behavioral Risk Factors**					
Current Cigarette Smoking					<0.001
Quintile 1	Reference	Reference	Reference	Reference	
Quintile 2	1.21 (1.20-1.22)	1.21 (1.20-1.22)	1.23 (1.22-1.23)	1.24 (1.23-1.24)	
Quintile 3	1.37 (1.35-1.38)	1.37 (1.36-1.39)	1.38 (1.37-1.39)	1.39 (1.38-1.40)	
Quintile 4	1.53 (1.51-1.55)	1.55 (1.53-1.56)	1.57 (1.56-1.59)	1.57 (1.56-1.59)	
Quintile 5	1.82 (1.80-1.85)	1.85 (1.83-1.87)	1.86 (1.84-1.88)	1.87 (1.85-1.90)	
No Leisure-Time Physical Activity					<0.001
Quintile 1	Reference	Reference	Reference	Reference	
Quintile 2	1.19 (1.18-1.20)	1.19 (1.18-1.20)	1.22 (1.22-1.23)	1.26 (1.25-1.27)	
Quintile 3	1.33 (1.32-1.34)	1.36 (1.35-1.37)	1.40 (1.39-1.41)	1.45 (1.44-1.47)	
Quintile 4	1.50 (1.49-1.51)	1.55 (1.53-1.56)	1.62 (1.61-1.63)	1.68 (1.66-1.69)	
Quintile 5	1.79 (1.77-1.81)	1.85 (1.84-1.87)	1.91 (1.90-1.93)	1.98 (1.95-2.00)	
**Cardiovascular Clinical Risk Factors**					
High Blood Pressure					<0.001
Quintile 1	Reference	Reference	Reference	Reference	
Quintile 2	1.07 (1.07-1.08)	1.07 (1.07-1.08)	1.09 (1.09-1.10)	1.11 (1.10-1.11)	
Quintile 3	1.13 (1.12-1.14)	1.14 (1.13-1.14)	1.15 (1.15-1.16)	1.18 (1.17-1.19)	
Quintile 4	1.19 (1.18-1.20)	1.20 (1.20-1.21)	1.23 (1.22-1.24)	1.27 (1.26-1.28)	
Quintile 5	1.29 (1.28-1.30)	1.31 (1.30-1.32)	1.33 (1.32-1.34)	1.37 (1.36-1.38)	
High Cholesterol					<0.001
Quintile 1	Reference	Reference	Reference	Reference	
Quintile 2	1.01 (1.01-1.02)	1.01 (1.01-1.02)	1.02 (1.02-1.03)	1.04 (1.03-1.04)	
Quintile 3	1.03 (1.03-1.04)	1.03 (1.03-1.04)	1.04 (1.03-1.04)	1.06 (1.05-1.06)	
Quintile 4	1.05 (1.04-1.06)	1.05 (1.04-1.05)	1.06 (1.05-1.06)	1.09 (1.08-1.10)	

© 2025 Lawrence WR et al. JAMA Health Forum.

Quintile 5	1.07 (1.06-1.08)	1.07 (1.07-1.08)	1.08 (1.07-1.08)	1.12 (1.11-1.13)	
Obesity					<0.001
Quintile 1	Reference	Reference	Reference	Reference	
Quintile 2	1.09 (1.08-1.10)	1.08 (1.08-1.09)	1.10 (1.09-1.10)	1.10 (1.10-1.11)	
Quintile 3	1.15 (1.14-1.16)	1.15 (1.14-1.16)	1.17 (1.16-1.17)	1.19 (1.18-1.19)	
Quintile 4	1.23 (1.22-1.23)	1.23 (1.23-1.24)	1.26 (1.25-1.27)	1.29 (1.28-1.30)	
Quintile 5	1.37 (1.36-1.38)	1.38 (1.37-1.39)	1.40 (1.39-1.41)	1.42 (1.41-1.43)	
Diabetes					<0.001
Quintile 1	Reference	Reference	Reference	Reference	
Quintile 2	1.15 (1.14-1.17)	1.15 (1.14-1.16)	1.18 (1.17-1.19)	1.23 (1.22-1.24)	
Quintile 3	1.27 (1.26-1.29)	1.29 (1.28-1.30)	1.32 (1.31-1.33)	1.39 (1.38-1.41)	
Quintile 4	1.43 (1.41-1.45)	1.46 (1.44-1.47)	1.52 (1.50-1.53)	1.60 (1.58-1.62)	
Quintile 5	1.73 (1.71-1.76)	1.77 (1.75-1.78)	1.80 (1.77-1.82)	1.90 (1.87-1.93)	
Cardiovascular Diseases					
Coronary Heart Disease					<0.001
Quintile 1	Reference	Reference	Reference	Reference	
Quintile 2	1.12 (1.10-1.13)	1.12 (1.11-1.13)	1.15 (1.14-1.15)	1.17 (1.16-1.18)	
Quintile 3	1.22 (1.20-1.24)	1.24 (1.23-1.25)	1.25 (1.24-1.27)	1.29 (1.28-1.31)	
Quintile 4	1.33 (1.31-1.35)	1.36 (1.35-1.38)	1.39 (1.38-1.41)	1.46 (1.44-1.47)	
Quintile 5	1.51 (1.49-1.53)	1.55 (1.53-1.57)	1.56 (1.54-1.59)	1.66 (1.63-1.69)	
Stroke					<0.001
Quintile 1	Reference	Reference	Reference	Reference	
Quintile 2	1.17 (1.15-1.19)	1.17 (1.16-1.18)	1.20 (1.19-1.21)	1.23 (1.22-1.24)	
Quintile 3	1.30 (1.29-1.32)	1.33 (1.32-1.35)	1.36 (1.35-1.37)	1.40 (1.38-1.41)	
Quintile 4	1.47 (1.45-1.49)	1.51 (1.50-1.53)	1.56 (1.54-1.57)	1.61 (1.60-1.63)	
Quintile 5	1.79 (1.77-1.82)	1.84 (1.82-1.86)	1.86 (1.84-1.89)	1.94 (1.91-1.96)	

[a] Multilevel linear mixed models weighted for population size and clustered at the county level, adjusting for age (median age), sex (percentage female), metropolitan status, region (Midwest, Northeast, Pacific, South, or West,), number of cardiovascular disease physicians per 100,000 population, number of primary care physicians per 100,000 population, percentage of adults having received a routine checkup in the last year, and percent uninsured adults. Estimates indicate increase in adjusted risk of cardiovascular risk factor and disease prevalence per unit increase in neighborhood structural racism

[b] Interaction between quintile structural racism and quartile of the percentage of Asian American population.

Note: Percentage Asian American Quartile Range (Quartile 1= ≤0.3%, Quartile 2= 0.3%-1.6%, Quartile 3= 1.6%-5.4%, Quartile 4= >5.4%)

Abbreviations: 95%CI, 95% confidence interval.

© 2025 Lawrence WR et al. JAMA Health Forum.

eTable 8. Adjusted Prevalence Rate Ratios for Cardiovascular Risk Factors and Cardiovascular Diseases by Neighborhood Structural Racism and Proportion of Black Population

	Percentage of Black Population in Quartile Adjusted Prevalence Risk Ratio (95%CI) [a]				
	First (lowest)	Second	Third	Fourth (Highest)	P interaction [b]
**Cardiovascular Behavioral Risk Factors**					
Current Cigarette Smoking					<0.001
Quintile 1	Reference	Reference	Reference	Reference	
Quintile 2	1.21 (1.20-1.22)	1.22 (1.21-1.22)	1.21 (1.20-1.22)	1.19 (1.17-1.20)	
Quintile 3	1.35 (1.34-1.36)	1.37 (1.36-1.38)	1.38 (1.37-1.39)	1.35 (1.33-1.37)	
Quintile 4	1.49 (1.48-1.51)	1.53 (1.52-1.55)	1.58 (1.56-1.59)	1.56 (1.54-1.58)	
Quintile 5	1.70 (1.68-1.73)	1.74 (1.71-1.76)	1.81 (1.79-1.83)	1.93 (1.90-1.95)	
No Leisure-Time Physical Activity					<0.001
Quintile 1	Reference	Reference	Reference	Reference	
Quintile 2	1.24 (1.23-1.25)	1.24 (1.24-1.25)	1.22 (1.21-1.23)	1.18 (1.17-1.19)	
Quintile 3	1.41 (1.40-1.42)	1.44 (1.43-1.45)	1.41 (1.40-1.42)	1.34 (1.32-1.35)	
Quintile 4	1.60 (1.59-1.61)	1.66 (1.65-1.68)	1.64 (1.63-1.65)	1.54 (1.52-1.56)	
Quintile 5	1.88 (1.86-1.90)	1.96 (1.94-1.98)	1.95 (1.93-1.97)	1.87 (1.85-1.89)	
**Cardiovascular Clinical Risk Factors**					
High Blood Pressure					<0.001
Quintile 1	Reference	Reference	Reference	Reference	
Quintile 2	1.09 (1.09-1.09)	1.10 (1.10-1.11)	1.09 (1.09-1.10)	1.07 (1.07-1.08)	
Quintile 3	1.15 (1.15-1.16)	1.18 (1.17-1.18)	1.17 (1.16-1.18)	1.13 (1.12-1.14)	
Quintile 4	1.22 (1.21-1.23)	1.27 (1.26-1.27)	1.26 (1.25-1.27)	1.21 (1.20-1.22)	
Quintile 5	1.31 (1.30-1.32)	1.38 (1.37-1.39)	1.39 (1.38-1.40)	1.32 (1.31-1.33)	
High Cholesterol					<0.001
Quintile 1	Reference	Reference	Reference	Reference	
Quintile 2	1.04 (1.04-1.04)	1.05 (1.04-1.05)	1.04 (1.04-1.05)	1.02 (1.01-1.03)	
Quintile 3	1.07 (1.07-1.08)	1.09 (1.08-1.09)	1.08 (1.07-1.08)	1.04 (1.03-1.05)	
Quintile 4	1.11 (1.10-1.11)	1.14 (1.13-1.14)	1.12 (1.12-1.13)	1.06 (1.05-1.07)	
Quintile 5	1.15 (1.15-1.16)	1.20 (1.19-1.20)	1.19 (1.18-1.20)	1.10 (1.09-1.11)	

© 2025 Lawrence WR et al. JAMA Health Forum.

Obesity					<0.001
Quintile 1	Reference	Reference	Reference	Reference	
Quintile 2	1.07 (1.06-1.07)	1.08 (1.08-1.08)	1.09 (1.08-1.09)	1.09 (1.08-1.10)	
Quintile 3	1.12 (1.11-1.12)	1.15 (1.14-1.15)	1.16 (1.15-1.17)	1.17 (1.16-1.18)	
Quintile 4	1.19 (1.18-1.19)	1.23 (1.22-1.24)	1.25 (1.24-1.25)	1.27 (1.26-1.28)	
Quintile 5	1.28 (1.27-1.29)	1.33 (1.32-1.34)	1.36 (1.35-1.37)	1.42 (1.41-1.43)	
Diabetes					<0.001
Quintile 1	Reference	Reference	Reference	Reference	
Quintile 2	1.19 (1.18-1.19)	1.20 (1.19-1.21)	1.18 (1.17-1.19)	1.14 (1.13-1.16)	
Quintile 3	1.33 (1.32-1.34)	1.37 (1.36-1.38)	1.34 (1.33-1.35)	1.27 (1.25-1.28)	
Quintile 4	1.49 (1.48-1.50)	1.57 (1.56-1.59)	1.55 (1.54-1.57)	1.44 (1.42-1.46)	
Quintile 5	1.77 (1.75-1.79)	1.88 (1.85-1.90)	1.88 (1.86-1.91)	1.77 (1.74-1.80)	
Cardiovascular Diseases					
Coronary Heart Disease					<0.001
Quintile 1	Reference	Reference	Reference	Reference	
Quintile 2	1.19 (1.18-1.19)	1.21 (1.20-1.21)	1.18 (1.17-1.19)	1.12 (1.10-1.14)	
Quintile 3	1.33 (1.32-1.34)	1.38 (1.37-1.39)	1.35 (1.34-1.37)	1.22 (1.20-1.24)	
Quintile 4	1.49 (1.48-1.50)	1.58 (1.57-1.60)	1.56 (1.54-1.57)	1.37 (1.34-1.39)	
Quintile 5	1.74 (1.72-1.76)	1.88 (1.86-1.90)	1.87 (1.85-1.89)	1.60 (1.57-1.64)	
Stroke					<0.001
Quintile 1	Reference	Reference	Reference	Reference	
Quintile 2	1.20 (1.20-1.21)	1.23 (1.22-1.23)	1.21 (1.20-1.22)	1.15 (1.13-1.17)	
Quintile 3	1.36 (1.35-1.37)	1.42 (1.40-1.43)	1.40 (1.39-1.41)	1.29 (1.27-1.31)	
Quintile 4	1.53 (1.52-1.54)	1.63 (1.62-1.65)	1.64 (1.62-1.65)	1.50 (1.47-1.52)	
Quintile 5	1.81 (1.79-1.84)	1.96 (1.93-1.98)	2.00 (1.98-2.03)	1.87 (1.84-1.90)	

[a] Multilevel linear mixed models weighted for population size and clustered at the county level, adjusting for age (median age), sex (percentage female), metropolitan status, region (Midwest, Northeast, Pacific, South, or West,), number of cardiovascular disease physicians per 100,000 population, number of primary care physicians per 100,000 population, percentage of adults having received a routine checkup in the last year, and percent uninsured adults. Estimates indicate increase in adjusted risk of cardiovascular risk factor and disease prevalence per unit increase in neighborhood structural racism

[b] Interaction between quintile structural racism and quartile of the percentage of Black population.

Note: Percentage Black Individual Quartile Range (Quartile 1= ≤1.0%, Quartile 2= 1.0%-4.3%, Quartile 3= 4.3%-15.6%, Quartile 4= <15.6%)

Abbreviations: 95%CI, 95% confidence interval.

© 2025 Lawrence WR et al. JAMA Health Forum.

eTable 9. Adjusted Prevalence Rate Ratios for Cardiovascular Risk Factors and Cardiovascular Diseases by Neighborhood Structural Racism and Proportion Hispanic or Latino Population

	Percentage of Hispanic or Latino Population in Quartile Adjusted Prevalence Risk Ratio (95%CI) [a]				
	First (lowest)	Second	Third	Fourth (Highest)	P interaction [b]
**Cardiovascular Behavioral Risk Factors**					
Current Cigarette Smoking					<0.001
Quintile 1	Reference	Reference	Reference	Reference	
Quintile 2	1.26 (1.25-1.27)	1.24 (1.23-1.25)	1.21 (1.20-1.22)	1.15 (1.14-1.16)	
Quintile 3	1.47 (1.46-1.48)	1.43 (1.42-1.44)	1.37 (1.36-1.39)	1.28 (1.27-1.29)	
Quintile 4	1.70 (1.68-1.71)	1.66 (1.65-1.68)	1.57 (1.56-1.59)	1.45 (1.43-1.46)	
Quintile 5	2.11 (2.09-2.14)	2.07 (2.05-2.10)	1.92 (1.90-1.94)	1.69 (1.68-1.71)	
No Leisure-Time Physical Activity					<0.001
Quintile 1	Reference	Reference	Reference	Reference	
Quintile 2	1.21 (1.20-1.22)	1.21 (1.20-1.22)	1.20 (1.19-1.21)	1.19 (1.18-1.20)	
Quintile 3	1.38 (1.37-1.39)	1.38 (1.37-1.39)	1.35 (1.34-1.36)	1.37 (1.36-1.38)	
Quintile 4	1.56 (1.55-1.58)	1.57 (1.56-1.58)	1.52 (1.51-1.54)	1.60 (1.58-1.61)	
Quintile 5	1.89 (1.88-1.91)	1.89 (1.87-1.91)	1.80 (1.78-1.82)	1.90 (1.89-1.92)	
**Cardiovascular Clinical Risk Factors**					
High Blood Pressure					<0.001
Quintile 1	Reference	Reference	Reference	Reference	
Quintile 2	1.09 (1.08-1.09)	1.09 (1.08-1.09)	1.08 (1.08-1.09)	1.08 (1.07-1.09)	
Quintile 3	1.16 (1.15-1.16)	1.15 (1.14-1.16)	1.14 (1.13-1.14)	1.15 (1.15-1.16)	
Quintile 4	1.23 (1.22-1.23)	1.22 (1.21-1.23)	1.20 (1.19-1.21)	1.25 (1.24-1.26)	
Quintile 5	1.33 (1.32-1.34)	1.32 (1.31-1.33)	1.30 (1.29-1.31)	1.37 (1.36-1.38)	
High Cholesterol					<0.001
Quintile 1	Reference	Reference	Reference	Reference	
Quintile 2	1.01 (1.01-1.02)	1.02 (1.01-1.02)	1.02 (1.01-1.02)	1.03 (1.02-1.03)	
Quintile 3	1.03 (1.03-1.04)	1.03 (1.03-1.04)	1.02 (1.01-1.03)	1.05 (1.05-1.06)	
Quintile 4	1.05 (1.05-1.06)	1.05 (1.04-1.06)	1.03 (1.02-1.04)	1.09 (1.08-1.10)	
Quintile 5	1.07 (1.07-1.08)	1.06 (1.05-1.07)	1.05 (1.04-1.05)	1.14 (1.13-1.14)	

© 2025 Lawrence WR et al. JAMA Health Forum.

Obesity					<0.001
Quintile 1	Reference	Reference	Reference	Reference	
Quintile 2	1.10 (1.10-1.11)	1.10 (1.09-1.10)	1.08 (1.08-1.09)	1.09 (1.08-1.09)	
Quintile 3	1.18 (1.17-1.18)	1.17 (1.16-1.17)	1.16 (1.15-1.17)	1.17 (1.17-1.18)	
Quintile 4	1.27 (1.26-1.28)	1.26 (1.25-1.26)	1.25 (1.24-1.26)	1.28 (1.27-1.29)	
Quintile 5	1.44 (1.44-1.45)	1.43 (1.42-1.44)	1.40 (1.39-1.41)	1.42 (1.41-1.43)	
Diabetes					<0.001
Quintile 1	Reference	Reference	Reference	Reference	
Quintile 2	1.16 (1.15-1.17)	1.16 (1.15-1.17)	1.16 (1.15-1.17)	1.18 (1.17-1.19)	
Quintile 3	1.30 (1.30-1.31)	1.29 (1.28-1.30)	1.27 (1.25-1.28)	1.35 (1.33-1.36)	
Quintile 4	1.47 (1.46-1.49)	1.45 (1.44-1.47)	1.41 (1.39-1.42)	1.57 (1.55-1.59)	
Quintile 5	1.80 (1.78-1.81)	1.74 (1.72-1.76)	1.67 (1.65-1.69)	1.91 (1.89-1.94)	
Cardiovascular Diseases					
Coronary Heart Disease					<0.001
Quintile 1	Reference	Reference	Reference	Reference	
Quintile 2	1.13 (1.12-1.14)	1.14 (1.13-1.15)	1.12 (1.11-1.13)	1.12 (1.10-1.13)	
Quintile 3	1.25 (1.24-1.27)	1.26 (1.24-1.27)	1.22 (1.20-1.23)	1.25 (1.23-1.26)	
Quintile 4	1.38 (1.37-1.39)	1.39 (1.37-1.40)	1.32 (1.31-1.34)	1.43 (1.41-1.45)	
Quintile 5	1.56 (1.55-1.58)	1.53 (1.51-1.55)	1.48 (1.45-1.50)	1.68 (1.65-1.70)	
Stroke					<0.001
Quintile 1	Reference	Reference	Reference	Reference	
Quintile 2	1.20 (1.19-1.21)	1.20 (1.19-1.21)	1.18 (1.17-1.19)	1.16 (1.15-1.18)	
Quintile 3	1.37 (1.36-1.39)	1.36 (1.34-1.37)	1.32 (1.30-1.33)	1.34 (1.32-1.35)	
Quintile 4	1.57 (1.56-1.58)	1.55 (1.53-1.56)	1.49 (1.47-1.50)	1.57 (1.55-1.59)	
Quintile 5	1.96 (1.94-1.98)	1.88 (1.86-1.91)	1.79 (1.77-1.81)	1.93 (1.91-1.96)	

[a] Multilevel linear mixed models weighted for population size and clustered at the county level, adjusting for age (median age), sex (percentage female), metropolitan status, region (Midwest, Northeast, Pacific, South, or West,), number of cardiovascular disease physicians per 100,000 population, number of primary care physicians per 100,000 population, percentage of adults having received a routine checkup in the last year, and percent uninsured adults. Estimates indicate increase in adjusted risk of cardiovascular risk factor and disease prevalence per unit increase in neighborhood structural racism

[b] Interaction between quintile structural racism and quartile of the percentage of Hispanic or Latino population.

Note: Percentage Hispanic or Latino Individual Quartile Range (Quartile 1= ≤2.8%, Quartile 2= 2.8%-7.6%, Quartile 3= 7.6%-20.9%, Quartile 4= >20.9%)

Abbreviations: 95%CI, 95% confidence interval.

© 2025 Lawrence WR et al. JAMA Health Forum.

eTable 10. Adjusted Prevalence Rate Ratios for Cardiovascular Risk Factors and Cardiovascular Diseases by Neighborhood Structural Racism and Proportion White Population

	Percentage of White Population in Quartile Adjusted Prevalence Risk Ratio (95%CI) [a]				
	First (lowest)	Second	Third	Fourth (Highest)	P interaction [b]
**Cardiovascular Behavioral Risk Factors**					
Current Cigarette Smoking					<0.001
Quintile 1	Reference	Reference	Reference	Reference	
Quintile 2	1.23 (1.22-1.25)	1.22 (1.21-1.23)	1.23 (1.22-1.24)	1.20 (1.19-1.20)	
Quintile 3	1.41 (1.39-1.42)	1.38 (1.37-1.39)	1.40 (1.39-1.41)	1.35 (1.34-1.36)	
Quintile 4	1.61 (1.60-1.63)	1.57 (1.56-1.59)	1.58 (1.57-1.60)	1.50 (1.48-1.51)	
Quintile 5	1.98 (1.96-2.00)	1.82 (1.80-1.84)	1.80 (1.78-1.81)	1.69 (1.67-1.71)	
No Leisure-Time Physical Activity					<0.001
Quintile 1	Reference	Reference	Reference	Reference	
Quintile 2	1.22 (1.21-1.23)	1.23 (1.22-1.24)	1.22 (1.21-1.23)	1.20 (1.19-1.20)	
Quintile 3	1.40 (1.39-1.42)	1.41 (1.40-1.42)	1.41 (1.40-1.42)	1.35 (1.34-1.36)	
Quintile 4	1.61 (1.60-1.62)	1.64 (1.63-1.65)	1.62 (1.61-1.63)	1.51 (1.50-1.52)	
Quintile 5	1.97 (1.95-1.99)	1.93 (1.91-1.94)	1.90 (1.88-1.92)	1.74 (1.73-1.76)	
**Cardiovascular Clinical Risk Factors**					
High Blood Pressure					<0.001
Quintile 1	Reference	Reference	Reference	Reference	
Quintile 2	1.11 (1.11-1.12)	1.11 (1.10-1.11)	1.10 (1.09-1.10)	1.08 (1.07-1.08)	
Quintile 3	1.20 (1.19-1.20)	1.18 (1.17-1.19)	1.17 (1.16-1.17)	1.14 (1.13-1.14)	
Quintile 4	1.29 (1.28-1.30)	1.27 (1.26-1.28)	1.25 (1.24-1.26)	1.20 (1.19-1.20)	
Quintile 5	1.42 (1.41-1.43)	1.38 (1.37-1.39)	1.35 (1.34-1.36)	1.28 (1.27-1.29)	
High Cholesterol					<0.001
Quintile 1	Reference	Reference	Reference	Reference	
Quintile 2	1.03 (1.02-1.04)	1.05 (1.04-1.05)	1.04 (1.04-1.05)	1.03 (1.03-1.04)	
Quintile 3	1.05 (1.05-1.06)	1.08 (1.07-1.08)	1.07 (1.07-1.08)	1.06 (1.06-1.07)	
Quintile 4	1.09 (1.08-1.09)	1.12 (1.11-1.13)	1.12 (1.11-1.12)	1.09 (1.09-1.10)	
Quintile 5	1.14 (1.13-1.14)	1.17 (1.16-1.18)	1.17 (1.16-1.18)	1.14 (1.14-1.15)	

© 2025 Lawrence WR et al. JAMA Health Forum.

Obesity					<0.001
Quintile 1	Reference	Reference	Reference	Reference	
Quintile 2	1.16 (1.15-1.17)	1.11 (1.10-1.11)	1.08 (1.07-1.08)	1.06 (1.05-1.06)	
Quintile 3	1.29 (1.28-1.30)	1.18 (1.17-1.19)	1.14 (1.13-1.14)	1.10 (1.10-1.11)	
Quintile 4	1.43 (1.42-1.44)	1.27 (1.27-1.28)	1.21 (1.20-1.22)	1.15 (1.15-1.16)	
Quintile 5	1.60 (1.59-1.61)	1.38 (1.38-1.39)	1.30 (1.29-1.31)	1.23 (1.22-1.23)	
Diabetes					<0.001
Quintile 1	Reference	Reference	Reference	Reference	
Quintile 2	1.19 (1.18-1.21)	1.20 (1.18-1.21)	1.17 (1.16-1.18)	1.15 (1.14-1.15)	
Quintile 3	1.36 (1.34-1.37)	1.34 (1.33-1.35)	1.32 (1.31-1.33)	1.27 (1.26-1.28)	
Quintile 4	1.56 (1.54-1.58)	1.56 (1.54-1.57)	1.51 (1.50-1.52)	1.41 (1.40-1.42)	
Quintile 5	1.92 (1.89-1.94)	1.85 (1.82-1.87)	1.78 (1.76-1.80)	1.63 (1.61-1.65)	
Cardiovascular Diseases					
Coronary Heart Disease					<0.001
Quintile 1	Reference	Reference	Reference	Reference	
Quintile 2	1.15 (1.13-1.17)	1.19 (1.18-1.21)	1.20 (1.19-1.21)	1.17 (1.17-1.18)	
Quintile 3	1.27 (1.26-1.29)	1.35 (1.33-1.36)	1.37 (1.36-1.38)	1.32 (1.31-1.33)	
Quintile 4	1.44 (1.42-1.46)	1.56 (1.54-1.57)	1.56 (1.55-1.58)	1.47 (1.46-1.48)	
Quintile 5	1.72 (1.70-1.75)	1.82 (1.80-1.85)	1.83 (1.80-1.85)	1.71 (1.69-1.73)	
Stroke					<0.001
Quintile 1	Reference	Reference	Reference	Reference	
Quintile 2	1.23 (1.21-1.24)	1.23 (1.22-1.24)	1.22 (1.21-1.23)	1.19 (1.18-1.20)	
Quintile 3	1.41 (1.40-1.43)	1.41 (1.39-1.42)	1.41 (1.40-1.42)	1.34 (1.33-1.35)	
Quintile 4	1.66 (1.63-1.68)	1.65 (1.63-1.67)	1.63 (1.61-1.64)	1.51 (1.50-1.52)	
Quintile 5	2.08 (2.06-2.11)	1.98 (1.95-2.01)	1.93 (1.90-1.95)	1.77 (1.75-1.79)	

[a] Multilevel linear mixed models weighted for population size and clustered at the county level, adjusting for age (median age), sex (percentage female), metropolitan status, region (Midwest, Northeast, Pacific, South, or West,), number of cardiovascular disease physicians per 100,000 population, number of primary care physicians per 100,000 population, percentage of adults having received a routine checkup in the last year, and percent uninsured adults. Estimates indicate increase in adjusted risk of cardiovascular risk factor and disease prevalence per unit increase in neighborhood structural racism

[b] Interaction between quintile structural racism and quartile of the percentage of White population.

Note: Percentage White Individual Quartile Range (Quartile 1= ≤59.8%, Quartile 2= 59.8%-80.6%, Quartile 3= 80.6%-91.9%, Quartile 4= >91.9)

Abbreviations: 95%CI, 95% confidence interval.

© 2025 Lawrence WR et al. JAMA Health Forum.

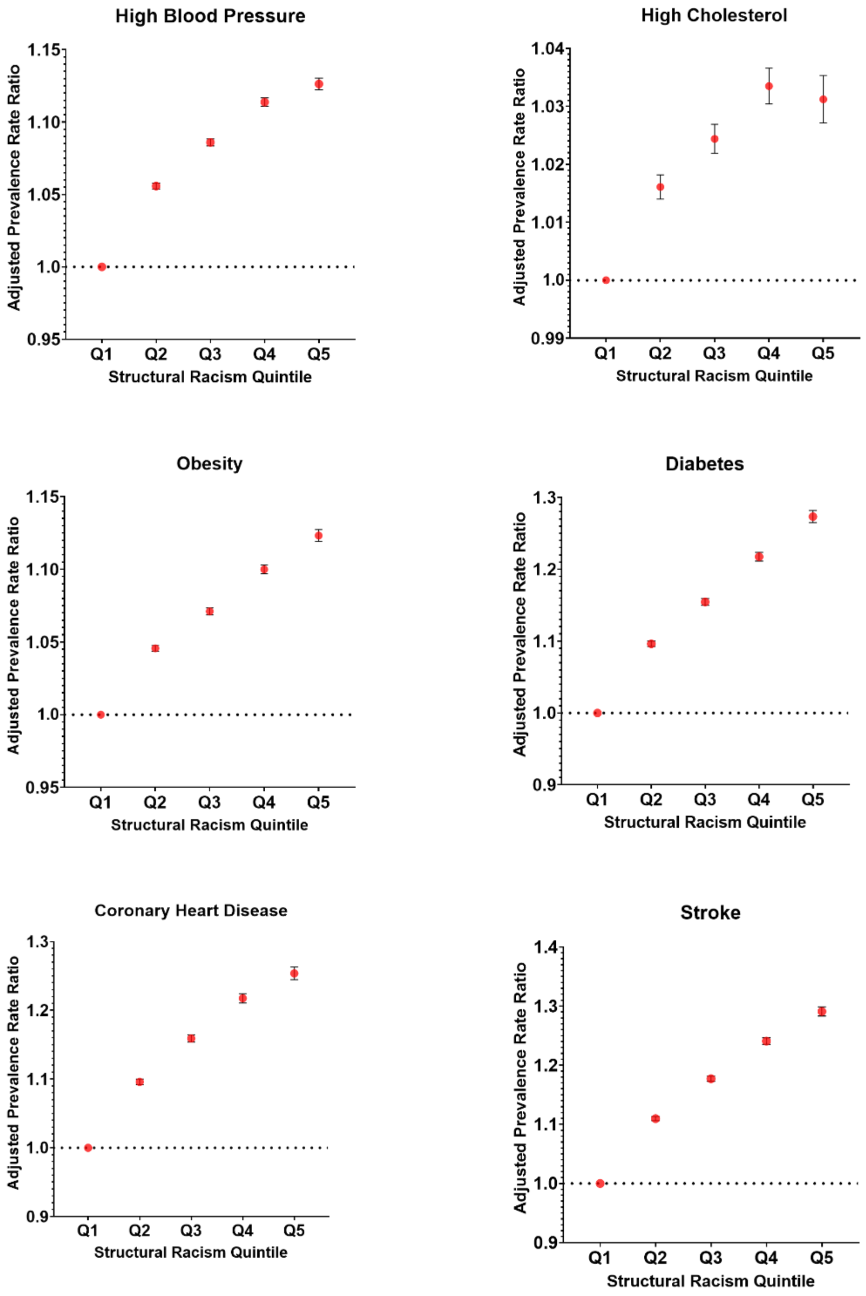

eFigure 2. Adjusted Prevalence Rate Ratios for Cardiovascular Clinical Risk Factors and Cardiovascular Diseases by Structural Racism Adjusting for Current Cigarette Smoking.

Multilevel linear mixed models weighted for population size and clustered at the county level, adjusting for age (median age), sex (percentage female), percentage of non-Hispanic White population, metropolitan status, region (Midwest, Northeast, Pacific, South, or West,), number of cardiovascular disease physicians per 100,000 population, number of primary care physicians per 100,000 population, percentage of adults having received a routine checkup in the last year, percent uninsured adults, and current cigarette smoking.

Q1=reference group.
Abbreviations Q=quintile
Note: Higher quintiles indicate greater negative effects of structural racism. Whiskers represent 95% CI.

© 2025 Lawrence WR et al. JAMA Health Forum.

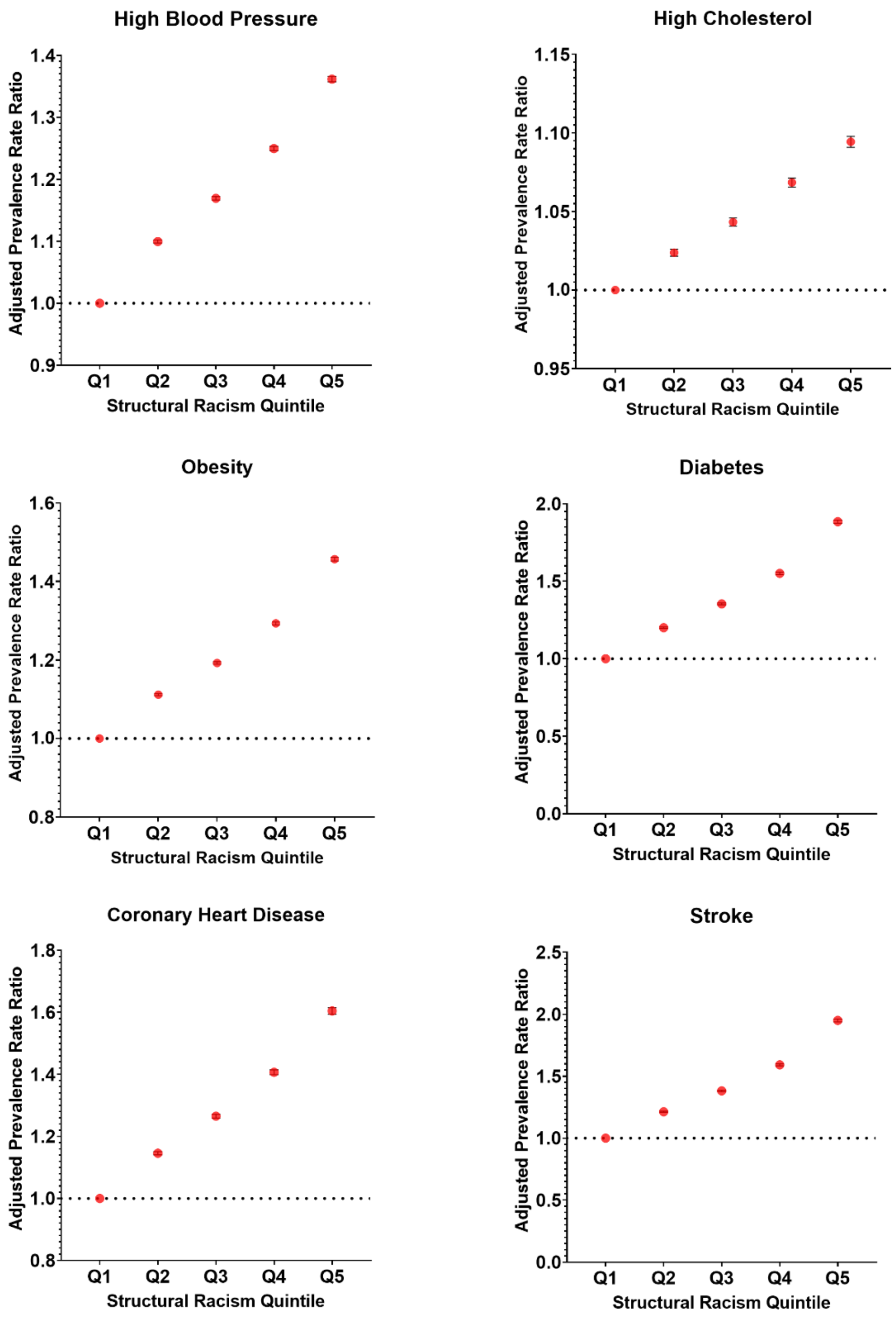

eFigure 3. Adjusted Prevalence Rate Ratios for Cardiovascular Clinical Risk Factors and Cardiovascular Diseases by Structural Racism, Not Adjusting for Percentage of Non-Hispanic White Population.

Multilevel linear mixed models weighted for population size and clustered at the county level, adjusting for age (median age), sex (percentage female), metropolitan status, region (Midwest, Northeast, Pacific, South, or West,), number of cardiovascular disease physicians per 100,000 population, number of primary care physicians per 100,000 population, percentage of adults having received a routine checkup in the last year, and percent uninsured adults.

Q1=reference group.
Abbreviations Q=quintile
Note: Higher quintiles indicate greater negative effects of structural racism. Whiskers represent 95% CI.

© 2025 Lawrence WR et al. JAMA Health Forum.

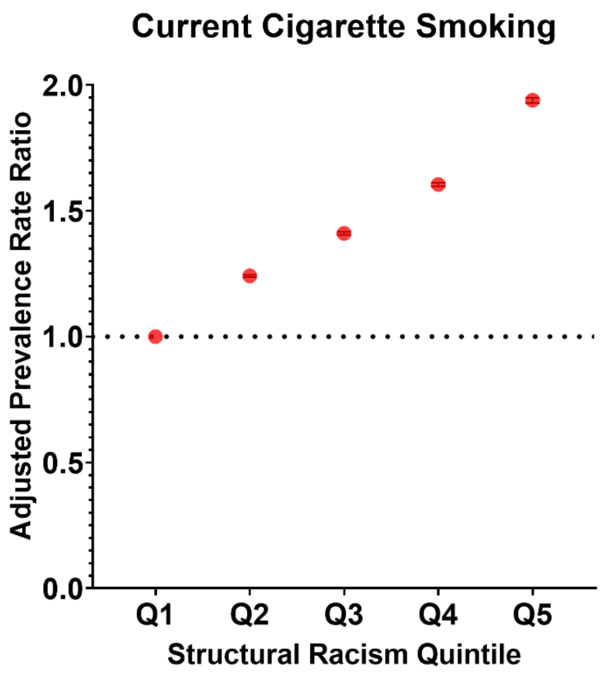

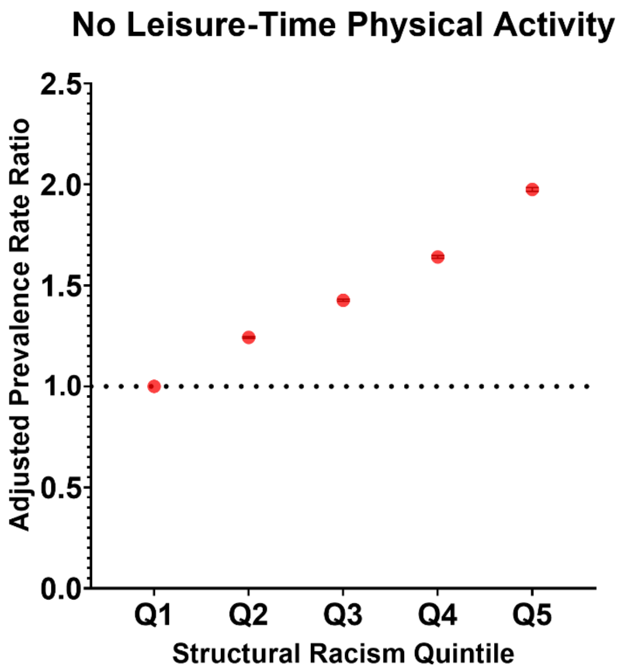

eFigure 4. Adjusted Prevalence Rate Ratios for Cardiovascular Behavioral Risk Factors by Structural Racism, Not Adjusting for Percentage of Non-Hispanic White Population.

Multilevel linear mixed models weighted for population size and clustered at the county level, adjusting for age (median age), sex (percentage female), metropolitan status, region (Midwest, Northeast, Pacific, South, or West,), number of cardiovascular disease physicians per 100,000 population, number of primary care physicians per 100,000 population, percentage of adults having received a routine checkup in the last year, and percent uninsured adults.

Q1=reference group.
Abbreviations Q=quintile
Note: Higher quintiles indicate greater negative effects of structural racism. Whiskers represent 95% CI.

© 2025 Lawrence WR et al. JAMA Health Forum.
