# Supplementary material for: Manifestations of Structural Racism and Inequities in Cardiovascular Health Across US Neighborhoods
Source: JAMA Health Forum. 2025 Oct 31;6(10):e253864. doi: 10.1001/jamahealthforum.2025.3864 (PMC12579350; doi:10.1001/jamahealthforum.2025.3864)
Supplement: Supplement 2. — Data Sharing Statement [file jamahealthforum-e253864-s002.pdf]

Data Sharing Statement

Lawrence. Manifestations of Structural Racism and Inequities in Cardiovascular Health Across US Neighborhoods. *JAMA Health Forum*. Published October 31, 2025. doi:10.1001/jamahealthforum.2025.3864

Data
Data available: Yes
Data types: Deidentified participant data
How to access data: CDC PLACES data can be found here: https://www.cdc.gov/places/measure-definitions/index.html and Structural Racism Effect Index data can be found here https://www.sreindex.com/background
When available: With publication

Supporting Documents
Document types: None

Additional Information
Who can access the data: anyone requesting the data
Types of analyses: for any purpose
Mechanisms of data availability: without investigator support
